# Improvement of Mutant Galactose-1-Phosphate Uridylyltransferase (GALT) Activity by FDA-Approved Pharmacochaperones: A Preliminary Study

**DOI:** 10.3390/ijms26030888

**Published:** 2025-01-21

**Authors:** Bernardina Scafuri, Stefania Piscosquito, Giulia Giliberti, Angelo Facchiano, Jaden Miner, Bijina Balakrishnan, Kent Lai, Anna Marabotti

**Affiliations:** 1 Department of Chemistry and Biology “A. Zambelli”, University of Salerno, 84084 Fisciano, Italy; bscafuri@unisa.it (B.S.);; 2 Institute of Food Science, National Research Council, 83100 Avellino, Italy; angelo.facchiano@isa.cnr.it; 3 Division of Medical Genetics, Department of Pediatrics, Spencer Fox Eccles School of Medicine, University of Utah, Salt Lake City, UT 84108, USAbijina.balakrishnan@hsc.utah.edu (B.B.); kent.lai@hsc.utah.edu (K.L.)

**Keywords:** drug repurposing, virtual screening, classic galactosemia, protein mutations, rare diseases, pharmacochaperones

## Abstract

Classic galactosemia is a rare disease with long-term consequences that seriously affect the quality of life of patients. To date, various therapeutic approaches are being developed, but treatments that target the molecular defects in the mutant galactose-1-phosphate uridylyltransferase (*GALT*) gene are lacking. We conducted a computational search for putative pharmacochaperones by applying a drug repurposing strategy, and we found that one compound, already active as a pharmacochaperone in another pathology, doubled the enzymatic activity of the purified mutant enzyme in an in vitro test. Furthermore, an extensive computational search in a database of known active molecules found another compound able in its turn to improve in vitro enzymatic activity. Both compounds are predicted to interact with a cavity at the enzyme interface previously supposed to be an allosteric site for the GALT enzyme. In vitro tests confirmed also the reduced accumulation of galactose-1-phosphate (G1P) in fibroblasts of patients. Although these results must be considered preliminary, our findings pave the way for future research lines focused on the search for promising pharmacochaperones that can directly rescue the activity of the enzyme.

## 1. Introduction

**Figure 1:**
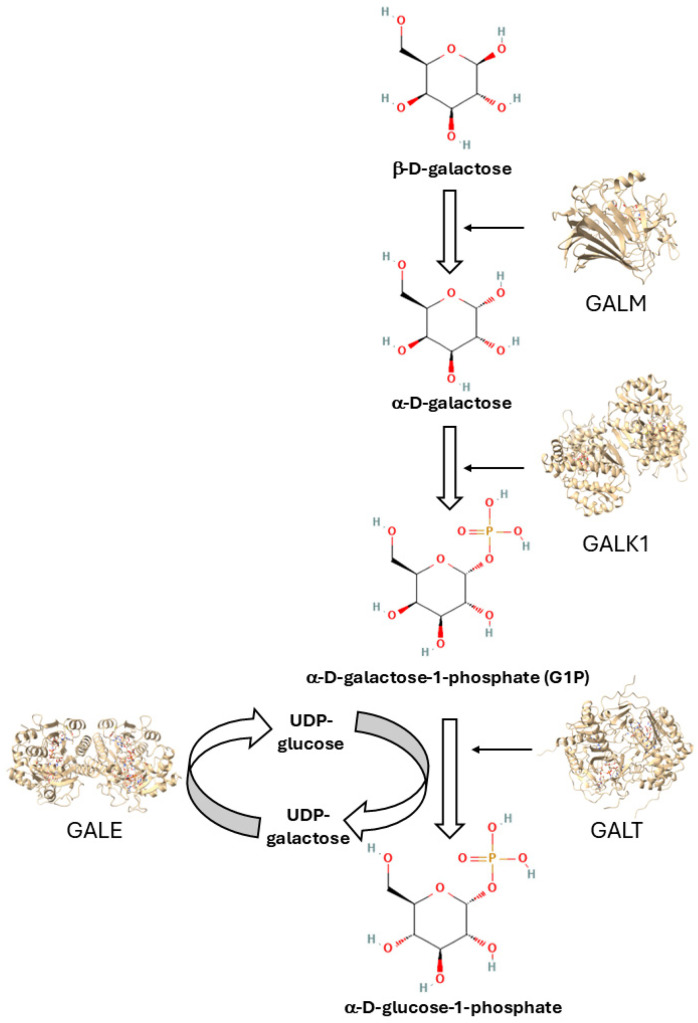
The Leloir pathway for galactose metabolism. This biochemical pathway is composed of four steps, each involving a different enzyme. In the first step, β-D-galactose is converted to α-D-galactose by the enzyme galactose mutarotase (GALM). Then, α-D-galactose is converted to galactose-1-phosphate (G1P) by galactokinase 1 (GALK1). Galactose-1-phosphate uridyltransferase (GALT) transfers a uridine monophosphate group from uridine diphosphate (UDP)-glucose to galactose-1-phosphate to form UDP-galactose. Finally, UDP-galactose-4′-epimerase (GALE) interconverts UDP-galactose and UDP-glucose. The figures of the sugars have been downloaded from PubChem (https://pubchem.ncbi.nlm.nih.gov/) (accessed on 12 January 2025), whereas the figures of the enzymes have been downloaded from the Galactosemia Proteins Database (http://protein-variants.eu/galactosemia/) (accessed on 12 January 2025) and rendered in cartoon mode with ChimeraX (https://www.rbvi.ucsf.edu/chimerax/) (accessed on 12 January 2025).

Classic galactosemia (CG) (OMIM #230400) is caused by the impairment of the enzyme galactose-1-phosphate uridyltransferase (GALT) (E.C. 2.7.7.12), one of the four enzymes that form the Leloir pathway for galactose catabolism [1], which is represented in Figure 1.

Mutations in the *GALT* gene [2] lead to the partial or total loss of this enzymatic activity, with both acute and chronic consequences in patients affected by this disease [3]. The lower the residual activity of the GALT enzyme, the greater the severity of the symptoms [4].

Research has long been striving to find effective therapies to alleviate the suffering of these patients. To reduce galactose-1-phosphate (G1P) levels, which is considered by many as a major culprit for long-term complications in CG, significant efforts have been deployed to develop inhibitors for the galactokinase 1 (GALK1) enzyme that converts galactose into G1P [5,6,7], but none of them are available for clinical use. Others have advocated the inhibition of the enzyme aldose reductase as a potential method to lower the elevated concentration of galactitol brought on by GALT impairment [8,9,10,11]. Other experimental treatments under investigation include the stimulation of cellular stress response [12], the intracellular delivery of exogenous GALT mRNA [13], and gene therapy [14,15,16]. Yet, despite promising preclinical results, only aldose reductase inhibitor AT-007 has reached the clinical trial stage.

As CG is caused by deleterious mutations in the *GALT* gene, which in turn have a negative impact on the structural dynamics and stability of the GALT enzyme [2,17,18,19,20], it is plausible to devise a therapeutic strategy based on the identification of pharmacological chaperones (PCs) that serve to augment the mutant GALT activity [21]. PCs are chemical compounds able to interact specifically with target proteins, stabilizing them against denaturation and thus rescue, at least partially, their activity [22,23]. The success of several PCs in restoring enzymatic activity and alleviating symptoms associated with several inherited diseases [24,25] prompted researchers to suggest this approach for CG in the past [18,26]. An attempt to use arginine (an aggregation inhibitor) to stabilize a mutant GALT enzyme appeared to rescue the activity of the enzyme in a prokaryotic model [27], but researchers failed to prove its effectiveness in a human trial [28], and arginine appeared not able to interact specifically with GALT at the molecular level [29].

Drug repurposing allows previously discovered and/or approved drugs to be re-used to treat new indications [30]. In fact, this approach has been successfully applied to identify promising PCs that have been used to stabilize mutant, non-functional enzymes involved in multiple genetic diseases [31]. To accelerate research and improve the rate of success, computational approaches can be used to perform in silico tests to validate the molecular interactions between the putative PCs and the target protein(s), thus establishing a rationale for their activity [32].

In this work, we present a computationally assisted drug repurposing approach to identify possible PCs for the most common and debilitating mutation associated with CG, p.Gln188Arg [33]. Docking approaches were applied to evaluate the likelihood that well-characterized PCs (ambroxol, pyrimethamine, and ciclopirox) could bind to the GALT enzyme, either at the active site or at other possible binding sites in meaningful manners, thereby stabilizing the mutant GALT enzyme. As such, ciclopirox was predicted to bind to a putative allosteric site of the enzyme near the intersubunit region [18,19]. A structure-based pharmacophore was derived from ciclopirox and used to make a virtual screening to search for other molecules capable of binding to the same pocket in the enzyme. Thus, another drug, ombrabulin, was predicted to interact with this dimeric protein in the same putative allosteric site of the enzyme. In vitro preliminary tests suggest that enzymatic activity might be improved by these drugs, thus providing a proof of principle of the validity of this approach to finding a new therapy for CG patients.

## 2. Results

### 2.1. Results by Computational Studies

#### 2.1.1. Docking Studies with Known PCs Selected from Literature

Computational methods can validly support the early stages of new drug discovery and drug repurposing, providing insights into promising molecules for disease treatment [32]. In the case of CG, our aim was to identify by computational methods possible PCs capable of stabilizing GALT, enhancing the activity in particular of the mutated form p.Gln188Arg.

**Figure 2:**
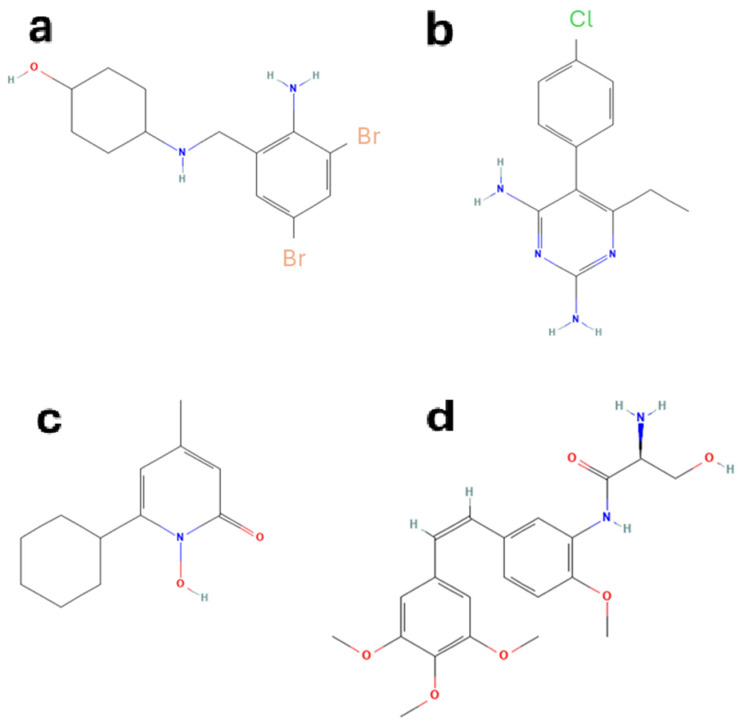
Chemical structures of the drugs tested in this work. Panel (**a**): ambroxol; panel (**b**): pyrimethamine; panel (**c**): ciclopirox; panel (**d**): ombrabulin. The first three molecules (panels (**a**–**c**)) were selected from the literature, as described in Section 2.1.1, whereas ombrabulin was identified by a virtual screening procedure based on the pharmacophore derived from ciclopirox, as described in Section 2.1.2. These structures have been downloaded from PubChem (https://pubchem.ncbi.nlm.nih.gov/) (accessed on 12 January 2025).

After an extensive and careful literature search, we selected for this preliminary investigation three FDA-approved drugs that have already been repurposed with proven efficacy as PCs. Their chemical structures are represented in Figure 2a–c. The first drug is ambroxol (trans-4-(2-amino-3,5-dibromobenzylamino)-cyclohexanol hydrochloride), a secretolytic agent commonly used in the treatment of respiratory diseases associated with viscid or excessive mucus [34], which was found able to enhance the activity of beta-glucocerebrosidase (GCase) [35,36] and to improve the progression of neurological symptoms in a clinical trial on Gaucher patients [37].

The second drug selected is pyrimethamine (5-(4-chlorophenyl)-6-ethylpyrimidine-2,4-diamine), an antiparasitic drug used as an antimalarial for its ability to inhibit dihydrofolate reductase [38], or together with a sulfonamide to treat toxoplasmosis [39]. This compound acts as a PC for beta-hexosaminidase (HEX_A) [40,41], an enzyme involved in the lysosomal storage disorders known as GM2-gangliosidosis, showing promising results in the treatment of these diseases [42].

Finally, we selected ciclopirox (6-cyclohexyl-1-hydroxy-4-methyl-2(1H)-pyridone), a compound with antifungal activity used for the treatment of several mycoses [43,44], which has been found to be able to bind and stabilize uroporphirinogen III synthase (UROS3), an enzyme involved in heme biosynthesis, whose impairment leads to a form of congenital erythropoietic porphyria [45].

First of all, we decided to perform docking studies using, for each drug, the respective protein target that was stabilized by that specific drug in the literature. The results are reported in Table S1. These results were used as a reference point for subsequent docking studies on GALT: results with a higher (worse) predicted binding energy and/or a higher number of clusters with respect to the known target for these drugs acting as PCs were considered less favorable interactions with the enzyme target of our investigation.

In order to identify in an unbiased way possible sites of interaction between the GALT enzyme (both the wild type (wt) and the mutant p.Gln188Arg form) and the selected drugs, we initially performed blind docking, i.e., by selecting the entire protein as a possible area of interaction. The results are shown in Tables S2 and S3 for wtGALT and p.Gln188Arg, respectively. Selected representative poses for each simulation are shown in Figure S1. For ambroxol and pyrimethamine, the energies of the best results in all conditions were higher (i.e., worse) than the energies of the interactions of these drugs with their target proteins reported in the literature, and associated with few poses, with a total number of clusters very high in both cases (higher in ambroxol than in pyrimethamine), indicating that probably these results do not identify any good interactions between GALT and these drugs. For both drugs, most poses belonging to the clusters with the best energy localized near either active site of the protein, contacting mainly residues belonging to only one chain, and frequently forming hydrogen bonds with the catalytic residue His186. Thus, these predicted interactions appear to have no particularly pronounced consequences for raising the stability of the protein, but they could hinder its catalytic activity. In addition, some spurious results (mostly associated to the most populated results in all conditions) revealed an interaction near the C-terminal of the protein chain, in a place where the interaction is probably not useful to stabilize the protein.

On the contrary, the poses identified in the simulations with ciclopirox in all conditions had the same or even better energy than the interaction of this drug with its published target protein, although the clusterization is not excellent. It is nonetheless similar to that of pyrimethamine, but better than that of ambroxol. The analysis of the interactions of ciclopirox showed that in all cases the preferred site for the interactions predicted by blind docking was near the active site, albeit not identical to the other two drugs. This compound is often predicted to interact simultaneously with residues belonging to both chains and only occasionally it is predicted to interact with the catalytic residue His186. Therefore, ciclopirox seems to have a better affinity for GALT than ambroxol and pyrimethamine (comparable to its known target), and in addition, its binding might be more able to raise the stability of the protein, with less negative impact on catalytic activity.

Focused docking simulations were then performed for each drug on both wtGALT and p.Gln188Arg, on a more restricted portion of the protein that includes the active site A. This type of docking allows us to refine the predicted interactions between each drug and this putative binding site on the GALT enzyme. The results are shown in Tables S4 and S5 for wtGALT and p.Gln188Arg, respectively. Selected representative poses for each simulation are shown in Figures S2 and S3. For both ambroxol and pyrimethamine, it is possible to note that, in the presence of only one of the two substrates, they tended to occupy the space left free by the other substrate both in wtGALT and p.Gln188Arg. Instead, when both substrates G1P and 5,6-dihydrouridine-5′-monophosphate (H2U) were in the active site, ambroxol and pyrimethamine located themselves either in the central cavity (poses with lower, i.e., better, energy) or at the surface of the protein (poses corresponding to the highly populated cluster). Finally, when no substrates were in the active site, ambroxol still identified the active site as the preferred place for binding, whereas pyrimethamine identified the active site for the pose with better energy, and the central cavity for the pose corresponding to the highly populated cluster. Looking at the predicted energies, they were significantly higher (i.e., worse) when both substrates were at the active site but were similar to those predicted by blind docking when only one of the two substrates was in the active site. In any case, the predicted energies were higher (i.e., worse) than those of the interactions of these drugs with their known target proteins, confirming that the interaction of these drugs with GALT was less favorable. The number of clusters was still relatively high when both substrates, or H2U alone, were in the active site, but decreased drastically when only G1P was present in the active site, or when no ligands were present, indicating that for both drugs, the preferred site for the interaction was the one occupied by H2U. Therefore, in all cases, forcing these drugs into the active site of GALT resulted in an apparently low specific interaction. Moreover, for both ambroxol and pyrimethamine, the predicted interaction with GALT seems to have interfered with the catalytic residue His186.

Ciclopirox interacted with wtGALT and p.Gln188Arg differently from the other two drugs: in all simulations it tended to bind preferably outside the active site (despite the simulation box being centered on the active site), frequently near the central cavity, with a similar energy to the one predicted by the blind docking simulations, and lower (i.e., better) than the one predicted for its known target. Also in this case, the interaction seems specifically to have identified a preferred zone at the interface between the two protomers composing the enzyme, suggesting that the binding of this molecule might improve the stability of the GALT enzyme.

We then set up a docking focused on this central cavity, which is important because it is made by residues belonging to both subunits, and therefore it could be an important point for the stabilization of the protein. The results are reported in Table S6 and representative poses for these simulations are shown in Figure S4. Also in this case, ambroxol and pyrimethamine showed interactions with worse predicted binding energies with respect to their known target proteins. In addition, ambroxol showed a high number of clusters, each populated by a low number of poses, indicating a low tendency to bind at this position, both in the absence and presence of the substrates (which, however, were outside the simulation box and therefore did not influence the results). Pyrimethamine showed a better clusterization, but still the predicted binding energy was worse than the one predicted for its known protein target, HEX_A. On the contrary, ciclopirox tended to occupy a different place in the central cavity with respect to ambroxol and pyrimethamine, and it showed a good predicted interaction energy (better than the one predicted for its known target), with a very high clusterization. This site appears to be a narrow zone of a bigger cavity identified also by FTMap as a “druggable” site, and looking at the literature, we found its position compatible with that of the “putative allosteric site” identified by McCorvie et al. [18], based on the analysis of the model of the GALT enzyme made available before the release of the crystallographic structure of GALT, on which the new models used in this study were based [2].

**Figure 3:**
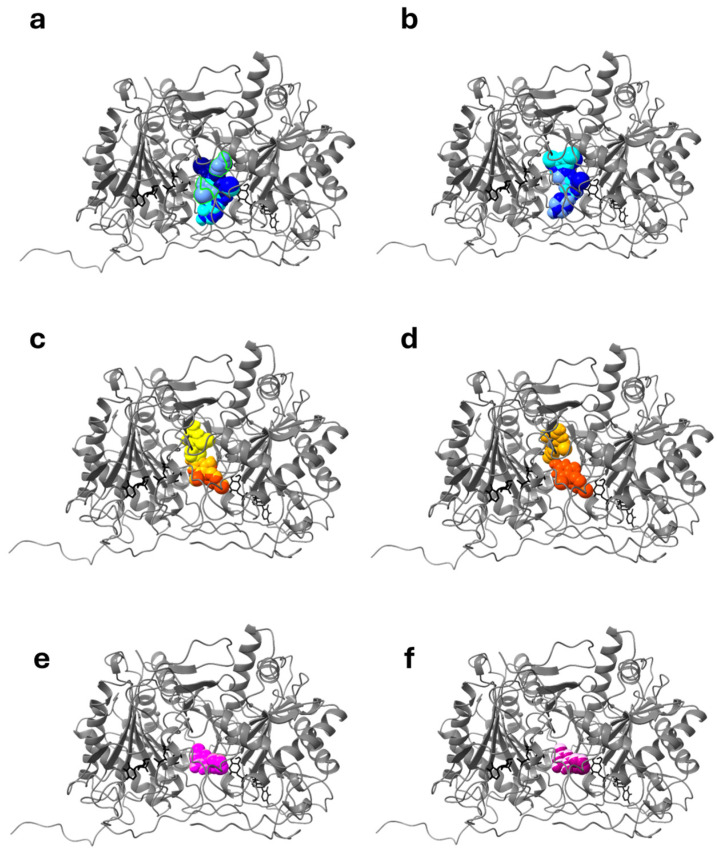
Interactions between wtGALT (**left** panels) with selected drugs, and between p.Gln188Arg enzyme (**right** panels) with selected drugs, identified by docking focused on the putative allosteric site (site X). All the docking simulations were made in the presence of both substrates in the active sites. Panels (**a**,**b**) show the docking of ambroxol in the box focused on site Xa (cyan) or Xb (blue); panels (**c**,**d**) show the docking of pyrimethamine in the box focused on site Xa (yellow) or Xb (orange); panels (**e**,**f**) show the docking of ciclopirox in the box focused on site Xa (pink) or Xb (magenta). The three drugs are represented in CPK mode. The substrates are represented in stick mode and colored black, the Zn ion as a sphere and colored grey. In contrast to ambroxol (panels (**a**,**b**)) and pyrimethamine (panels (**c**,**d**)), ciclopirox (panels (**e**,**f**)) identified only one conformation within the X site, thus showing a higher specificity of interaction with that site.

To explore these results further, we analyzed in detail the interactions of the three compounds with this specific putative binding site (which we called “site X”) of the protein. We performed focused docking using two boxes, partially overlapping, in order to better sample this zone. The results are reported in Table 1 and in Figure 3, which shows the most representative results (i.e., the poses characterized by the lowest energy and/or belonging to the most populated clusters) of these simulations for the three drugs.

**Figure 4:**
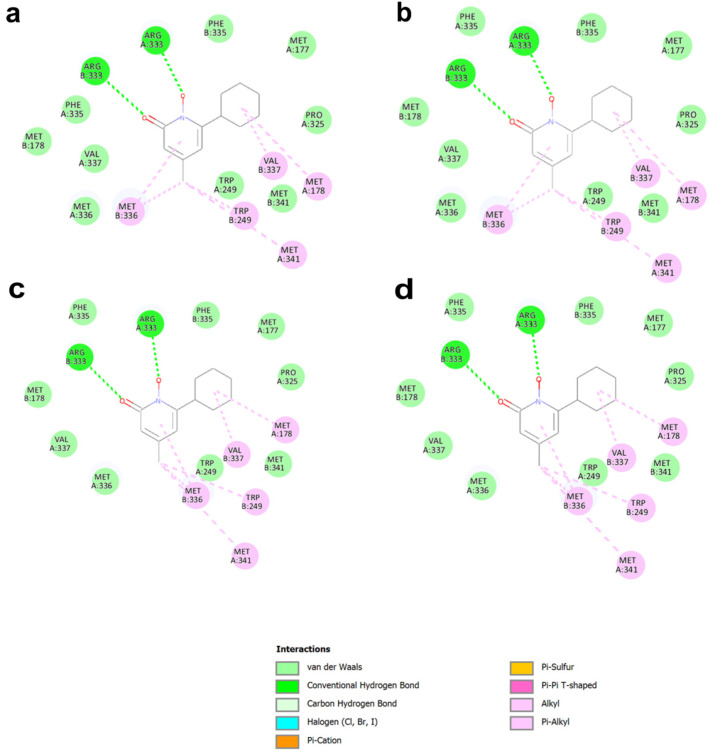
Detailed interactions between ciclopirox and wtGALT or p.Gln188Arg enzymes identified by docking focused on the putative allosteric site (site X). In all cases, the pose with best energy is also representative of the most populated cluster. Panel (**a**): result of docking on site Xa on wtGALT. Panel (**b**): result of docking on site Xa on p.Gln188Arg. Panel (**c**): result of docking on site Xb on wtGALT; Panel (**d**): result of docking on site Xb on p.Gln188Arg. In all cases, the interactions of ciclopirox with the enzyme were the same, confirming that the molecule binds specifically to the enzyme pocket.

The results of this focused docking show that ciclopirox bound the putative allosteric site (site X), which is in a zone of the protein in which the two boxes used for simulations overlapped, with a very high clusterization (99 to 100 poses) and with a favorable interaction energy, better than that of its known target. On the contrary, the results obtained by forcing ambroxol and pirimethamine to dock to this site were not equally favorable: either the clusterization was not unique, as in the case of ambroxol, or the binding affinity was less favorable, as in the case of pyrimethamine (Table 1), indicating that these two drugs are probably not likely to interact with this putative binding site as favorably as does ciclopirox. The analysis of the interactions between these three drugs and site X is reported in Figure 4 for ciclopirox and in Figures S5 and S6 for ambroxol and pyrimethamine, respectively. It is important to note that residues belonging to both chains interacted with the drug. In particular, for ciclopirox (Figure 4), Arg333 of both chains formed hydrogen bonds, whereas Met178 and Met341 of chain A formed alkyl and p-alkyl interactions, as did Trp249, Met336, and Val337 of chain B. Furthermore, van der Waals interactions are formed by Met177, Trp249, Pro325, Phe335, Met 336, and Val337 of chain A, and Met178, Phe335, and Met341 of chain B. Overall, this suggests that ciclopirox might stabilize the structure of GALT with an interaction characterized by a predicted binding energy more favorable than that with its known target enzyme, UROS3, and with a unique cluster of interactions that involve several residues at the interface between the two chains, in a very specific zone of the enzyme where no interference with the catalytic activity is anticipated. On the contrary, the interaction of ambroxol and pyrimethamine within the same specific zone was energetically less favorable than that predicted for their known targets, and the very scattered results indicate poor specificity of these two drugs toward this cavity.

Therefore, our computational approach was able to predict that of the three drugs initially tested as possible PCs for GALT, ciclopirox has the highest probability of success, as it is predicted to bind specifically to a cavity of the enzyme other than the active site, with good affinity and high clustering.

#### 2.1.2. Pharmacophore Screening for Further Selection of Putative Drugs

**Figure 5:**
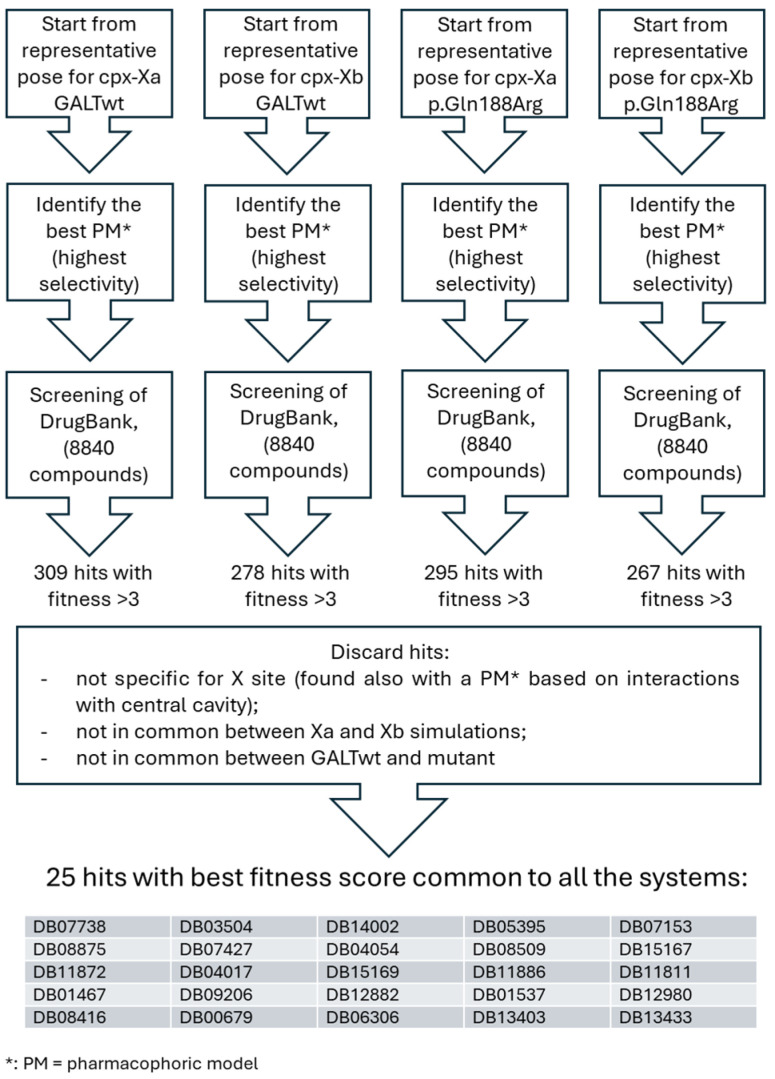
Further identification of pharmacological hits specific to site X of wtGALT and p.Gln188Arg enzymes starting from ciclopirox. Pharmacophoric models (PM) were obtained for each best pose of the docking of ciclopirox with site Xa and Xb. For each of these starting poses, the PM with the highest selectivity was used to make the corresponding pharmacophore hit search in DrugBank. Only hits with a fitness score > 3 (as recommended) were selected. Then, selection criteria were applied as described in the scheme, to identify the final 25 best hits.

Given the favorable interactions between ciclopirox and site X, we decided to perform a structure-based pharmacophore analysis starting from this interaction, in order to extract a pharmacophore model that could be useful to find other promising ligands for the GALT enzyme in that site. The pharmacophoric model with the highest selectivity was then used to perform a search in the DrugBank database, through Discovery Studio. In order to filter out those results that were not specific to site X, we performed similarly a pharmacophore analysis based on the interactions between ciclopirox and the more general results on the central cavity, and excluded all the hits that were common both to the central cavity and site X. We also excluded pharmacological hits that were found with pharmacophores obtained only from the poses of wtGALT or the mutant enzyme. After this selection, we analyzed the best 30 pharmacological hits for simulations on site X of wtGALT and of the mutant, and discarded those hits that were not common among these four lists. The scheme of this process is shown in Figure 5, together with the final list of 25 final pharmacological hits selected for further steps.

**Figure 6:**
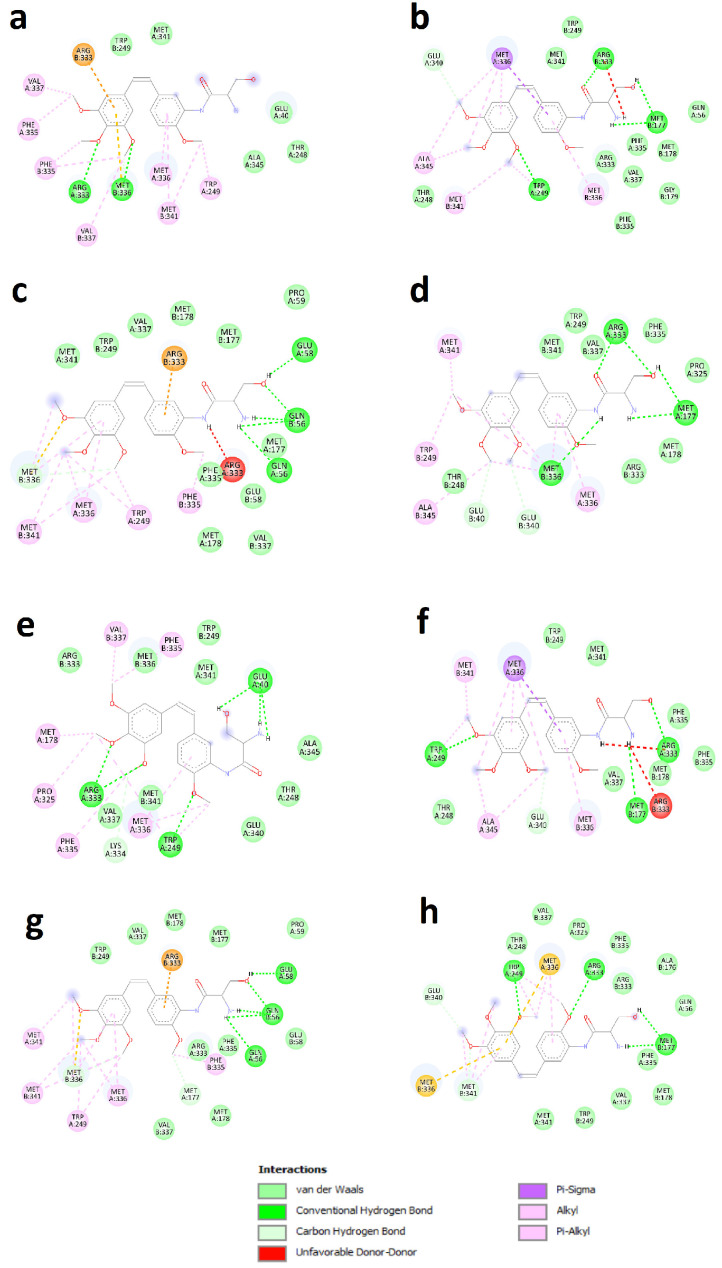
Detailed interactions between DB12882 (ombrabulin) and wtGALT or p.Gln188Arg enzymes identified by docking focused on the putative allosteric site (site X). Panel (**a**): result of docking on site Xa on wtGALT, pose with best energy. Panel (**b**): result of docking on site Xa on wtGALT, pose representative of the most populated cluster. Panel (**c**): result of docking on site Xb on wtGALT, pose with best energy. Panel (**d**): result of docking on site Xb on wtGALT, pose representing the most populated cluster. Panel (**e**): result of docking on site Xa on p.Gln188Arg, pose with best energy. Panel (**f**): result of docking on site Xa on p.Gln188Arg, pose representative of the most populated cluster. Panel (**g**): result of docking on site Xb on p.Gln188Arg, pose with best energy. Panel (**h**): result of docking on site Xb on p.Gln188Arg, pose representing the most populated cluster. The interactions between DB12882 (ombrabulin) and the putative allosteric site X involve most of the residues interacting with ciclopirox, thereby confirming the predicted ability of ombrabulin to interact with site X similarly to the parent compound ciclopirox, especially in the poses with the best energy.

When we re-docked the hits on the site X of both wtGALT and mutant enzyme, we ran into a problem with DB11872 in the setting of the simulations and therefore we excluded it from simulations. We used AutoDock and the same settings fixed for the previous compounds. The results are shown in File S1. We found that some pharmacological hits (selected among those classified by DrugBank as “approved” or “investigational”) showed a very favorable predicted binding energy and a favorable clusterization: in particular, on the basis of these data, DB06306, DB08875, DB12882, DB13403, and DB14002 were considered the five best. Figure 6 shows the interactions of DB12882 (ombrabulin, whose chemical structure is reported in Figure 2d) with the site X of both wtGALT and p.Gln188Arg; the interactions for the other compounds are reported in Figures S7–S10. In all cases, the interactions between these pharmacological hits and the sites were very similar to those of ciclopirox, indicating that these five compounds also could be promising PCs for this protein.

### 2.2. Results of Preliminary Experimental Assays

#### 2.2.1. In Vitro Evaluation of GALT Enzymatic Activity Augmentation by the Selected Small Molecules

**Figure 7:**
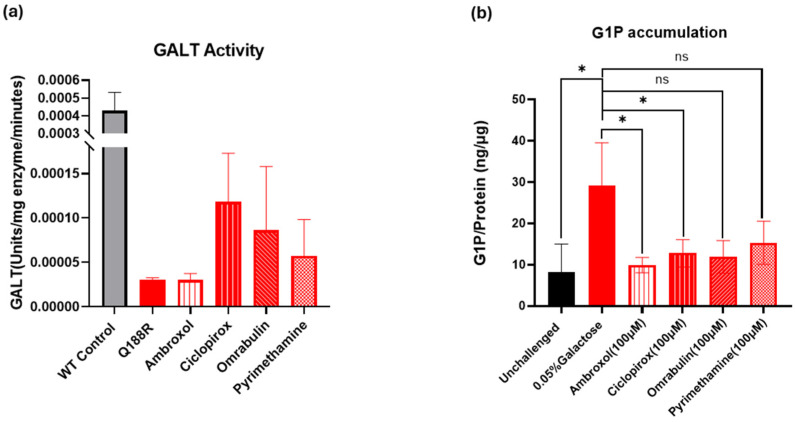
Effect of putative PCs on purified mutant GALT enzyme activity and galactose-1-phosphate (G1P) accumulation in patient fibroblasts. Panel (**a**): Purified mutant GALT enzyme activity was determined in the presence of different compounds. Enzyme activity is expressed as units/mg enzyme/minute. Error bars indicate mean activity ± standard deviation of the mean (n = 3 independent experiments). Panel (**b**): G1P levels detected in untreated wt, untreated patient, and patient fibroblasts treated with various compounds. Error bars represent mean G1P/protein concentrations ± standard deviation (n = 3 independent experiments. Statistical significance: * *p* < 0.05, compared to untreated patient fibroblasts (Student’s *t*-test). ns: not significant.

We incubated selected compounds (ambroxol, pyrimethamine, ciclopirox, and DB12882, a.k.a. ombrabulin) at 100 μM with purified mutant p.Gln188Arg GALT protein for 5 min and measured the GALT enzyme activity in their presence thereafter using the enzyme-linked activity assay for GALT described in Materials and Methods. The results were compared to wtGALT enzyme activity. Figure 7a summarizes the preliminary findings. In line with our predictions, the results show that ciclopirox doubles p.Gln188Arg GALT activity (up to 27% of wt value), followed by ombrabulin (up to 20.4% of wt value), pyrimethamine (up to 13% wt) and ambroxol (up to 7% wt). These results support our hypothesis that ciclopirox and ombrabulin can act as PCs for the mutant form of the GALT enzyme, thereby partially rescuing its enzymatic activity.

#### 2.2.2. Cell-Based Assessment of GALT Enzymatic Activity Augmentation by the Selected Small Molecules

As shown in Figure 7b, all three compounds at 100 μM reduced G1P accumulation at varying degrees, suggesting that they somehow improved galactose metabolism. Nevertheless, out of the three compounds tested, ambroxol showed the highest level of G1P reduction (~65%), followed by ombrabulin (~56.7%) and ciclopirox (~50%), and finally pyrimethamine (~45%). These results strengthen the hypothesis that these compounds can reduce the amount of toxic metabolite that accumulates as a result of impairment of the activity of the mutant GALT enzyme, thus reinforcing the prediction of their efficacy as PCs.

## 3. Discussion

Since their discovery [21], PCs have been considered an interesting pharmaceutical approach for the treatment of rare genetic diseases, which are often caused by mutations that destabilize enzymes and consequently impair their activity. However, identifying specific PCs able to exert a stabilizing activity on a selected target protein is not trivial. Computational approaches can help in this process, either by identifying cavities useful for the binding of PCs or by performing virtual screenings of compounds. Moreover, if the structure of the enzyme is known, it is possible to obtain information that can allow a more rational approach based on the interactions between the compounds and the target proteins [32]. In fact, many PCs currently available to treat different rare diseases were obtained by repurposing drugs currently in clinics for different therapeutic applications [22,23,31].

CG can be considered a “misfolding disease” due to the destabilizing effects of many mutations of the GALT enzyme [2,18,19], and several researchers have highlighted in the literature the opportunity to identify PCs that can be used as therapies for this disease [1,10,11,18,19,26]. p.Gln188Arg, the most common and debilitating mutation associated with this disease [3], affects a residue in the GALT active site and therefore alters its enzymatic activity. Yet, it can also exert a destabilizing effect on the protein, since this mutation is at the interface between the two subunits, and the introduction of a positive charge in a zone of the protein already rich in positively charged residue can cause a repulsion in the structure, leading to misfolding [19]. Since even a little residual activity of the enzyme can result in a considerable improvement in patient outcomes, a PC successfully targeting this mutant could significantly improve the quality of life of most galactosemic patients.

In this study, we employed an extensive computational approach to not only identify promising PCs but also infer the molecular mechanisms of action for these compounds. Ambroxol and pyrimethamine were predicted to bind non-specifically to the structure of GALT, targeting the substrates’ binding site and also other pockets in the protein. On the contrary, ciclopirox seems to find a specific binding site in the enzymatic structure. In particular, the most preferred one is a pocket at the dimer interface, already identified in the past [18] and considered as a putative “allosteric site” for the protein. At present, there is no clear evidence that this pocket is actually involved in allosteric pathways in the protein (computational studies to predict such pathways are currently ongoing); however, since this pocket consists of residues of both subunits (see Figure 4) and is not involved in enzyme activity, it appears to be a perfect target for the development of so-called second-generation PCs, whose stabilizing action on proteins does not involve interaction with substrate binding sites [32]. For this reason, we hypothesized that other compounds able to target the same pocket would be, in their turn, promising PCs for this enzyme, and we focused further research on identifying other compounds with promising binding affinity and interactions with proteins similar to this last compound. Therefore, we extracted a structure-based pharmacophore from this interaction, and we found that several compounds in DrugBank can share with ciclopirox the same features, being in their turn promising compounds to act on this protein.

We performed preliminary in vitro assays on the purified wtGALT and p.Gln188Arg, and we found that, indeed, ciclopirox and one of the derivatives obtained by the pharmacophoric search, DB12882 (ombrabulin), were able to improve the activity of the p.Gln188Arg mutant of the GALT enzyme. Of course, given the high concentrations tested, it is unlikely that these two compounds can be used directly as drugs to treat CG. Nevertheless, this proof of principle could be considered as a starting point either for the search for further compounds, already available, or for the improvement of the pharmacological properties of these molecules.

We also found that the tested compounds were able to reduce the G1P accumulation in fibroblasts derived from a CG patient, thereby improving their galactose metabolism. At this stage, it is difficult to understand whether this effect is exclusively due to the improvement in the activity of the GALT enzyme or to other collateral effects such as changes in galactokinase I activity or galactose transport.

More experiments will be necessary to advance this knowledge, but this encouraging results should justify other pharmacological strategies that could alleviate both the acute and long-term complications of CG patients.

## 4. Materials and Methods

### 4.1. Computational Approaches

The structures of the selected drugs used for this study were downloaded from DrugBank [46] in 3D conformation and saved in .pdb format. The structures of the proteins to which these drugs exert their activity as PCs were downloaded from PDB [47] (PDB codes: 1OGS for GCase; 2GK1 for HEX_A and 1JR2 for UROS3) and used as a reference for the evaluation of the relative affinity of the binding of their PCs with respect to the predicted affinity for GALT. In the case of GCase, the simulations were performed on chain A. The residues with an occupancy value of 0.50 (Q57, Q73, E151, D358, E388) were split in order to analyze both conformations.

The structural models of the dimeric wtGALT enzyme and of the mutant p.Gln188Arg enzyme, both with bound G1P and H2U, as well as the Zn^2+^ ions, were downloaded from the Galactosemia Proteins Database [2]. The search for the putative allosteric site, previously identified by McCorvie and colleagues [18,19], was performed on the two models used in the present work using FTMap Web server [48].

Docking studies were performed with AutoDock version 4.2.6, after preparing the system with MGLTools version 1.5.7 [49]. Polar hydrogens were added to the proteins and ligands (except for phosphate groups in ligands, which were considered deprotonated), and charges were assigned according to Gasteiger [50].

To test the interaction between GCase and ambroxol, a docking focused on the active site of GCase (including residues R120, W179, N234, E235, Y313, E340, and W381) was performed, setting up a grid of 64 × 54 × 40 points, with a default spacing of 0.375 Å. A focused docking strategy on the known binding site for pyrimethamine on HEX_A [51] (including R178, D207, H262, D322, E323, W373, Y421, N423, E462, and W470) was applied by setting up a grid of 58 × 68 × 52 points with a default spacing of 0.375 Å. Finally, for a docking focused on the allosteric site of UROS3 target of ciclopirox [45], a grid of 40 × 48 × 54 points (including S95, V96, Y97, D113, E115, T118, and Y128), with a default spacing of 0.375 Å, was set up.

Docking studies on the GALT enzyme (both wtGALT and p.Gln188Arg) were performed in different conditions, depending on the type of investigation made. For blind docking, a grid map with a spacing of 0.642 Å and dimensions of 110 × 100 × 120 points was set up to include the whole proteins. Simulations were made either with both substrates in the active sites, or by alternatively keeping G1P and H2U in the active sites, or by removing both ligands from the active sites. For docking focused on the active site of GALT formally identified as “A” (containing His186 of the chain A, as reported in the PDB file), a grid map of 58 × 80 × 74 points and a default spacing of 0.375 Å was set up. For each system, simulations were made either with both substrates in active site A, or by alternatively keeping G1P and H2U in active site A, or by removing both ligands from active site A; instead, active site B was left with both ligands.

For docking focused on the central cavity, a grid map of 68 × 86 × 72 points and a default spacing of 0.375 Å was set up. For these systems, simulations were made either with both substrates in the active sites, or by removing both substrates from the active sites. Docking focused on the putative allosteric site (site X) was made using two different but partially overlapping grid maps focused on Glu40 belonging either to chain A and B, in order to better map this site. These two grids had slightly different dimensions as the residues identifying this site are not perfectly specular in the protein. The dimension of the grid maps were 54 × 68 × 46 for the grid centered on Glu40 A and 56 × 56 × 62 for the grid centered on Glu40 B, both with default spacing of 0.375 Å. In both cases, ligands were left in the active sites, since they were not included in the grids.

For all docking simulations, 100 docking runs were performed using the AutoDock Lamarckian genetic algorithm, treating the protein as rigid and the ligand as flexible. All the other parameters were kept as default (population size: 150; number of energy evaluations: 2,500,000; and number of generations: 27,000). The docking poses were clustered using an RMSD value of 2.0 Å. The conformations representative of the best energetic and of the most populated clusters of poses were selected, saved in .pdb format, and analyzed for their interactions with the respective enzymes using DiscoveryStudio v. 4.5.1 (Biovia-Dassault Systèmes, San Diego, CA, USA), which was also used to perform a pharmacophoric search starting from the docking poses selected for each ligand, using default parameters. Of the models generated, we always selected the one with the highest selectivity (when the selectivity was the same for different models, all were considered). We used the models to perform a search for pharmacophoric hits in the DrugBank database, through DiscoveryStudio. For each generated pharmacological hit list for each model, we selected only those drugs with fit value ≥ 3. In addition, we considered the status of the drug (approved; experimental; investigational) and the compliance with Lipinski’s rule of five [52].

### 4.2. Preliminary Experimental Assays

Ambroxol hydrochloride (analytical standard), pyrimethamine (United States Pharmacopeia Reference Standard), and ciclopirox (HPLC grade) were purchased from Merck (Pennsylvania, PA, USA), whereas ombrabulin hydrochloride (compound DB12882 in DrugBank) was purchased from DC Chemicals Ltd. (Shanghai, China). All were of the highest purity grade available and were used without further purification.

GALT enzymatic assays were performed using the method described by Mayes and Hanson [53], with minor modifications. Briefly, recombinant human GALT enzyme was prepared as described by Lai et al. [54]. Purified wtGALT enzyme was used as a positive control. Assays for the overall double displacement reaction were carried out at 37 °C in 200 μL of glycine buffer (100 mm, pH 8.7) containing 0.6 mm UDP-Glu, 5 mm MgCl_2_, 5 mm dithiothreitol, 0.8 mm NADP, 1.2 mm G1P, 5 μm glucose-α-1,6-diphosphate, phosphoglucomutase (0.5 IU/mL), glucose-6-phosphate dehydrogenase (0.5 IU/mL), and recombinant GALT enzyme with or without specific small molecule compounds. The formation of NADPH was quantitated by absorbance change at 340 nm. The quantitative relationship between increase in NADPH production and glucose-1-phosphate released was quantitated using the Beer–Lambert equation: Absorbance = Concentration of solutes × Optical path length × Molar coefficient of extinction. In this study, the solute was NADPH, the optical path length was 1 cm, and the molar coefficient of extinction for NADPH at 340 nm was 6220 M^−1^ cm^−1^. We calculated ΔAbs 340 (change in the absorbance) = ΔC (change in the concentration) × 1 × 6220. All biochemicals and enzymes required for the GALT assay were purchased from Merck (Pennsylvania, PA, USA).

Primary fibroblast cells (GM01703) derived from a patient who is homozygous for the p.Gln188Arg mutation were used in this assay. Unchallenged cells and galactose-challenged cells were used as baseline and maximum controls for galactose-1-phosphate accumulation. Cells were obtained from the Coriell Cell Repository through a materials transfer agreement between the University of Utah and the repository. Cultured cells in normal DMEM + 10% FBS medium were incubated with the selected compounds at 100 μM for 16 h prior to exogenous 0.05% galactose challenge for 4 h. At the end of the challenge, cells were harvested and analyzed for G1P, a galactose metabolite that accumulates in GALT deficiency.

## 5. Conclusions

Drug repurposing is an effective strategy to reduce the overall time and costs often needed in drug development. In the present work, using a computational approach, we extended the potential use of several clinically approved pharmacochaperones for other diseases to classic galactosemia. Our analyses indicated that ciclopirox specifically bound to a pocket at the dimeric interface of the enzyme without interfering with its enzymatic activity, and this interaction could strengthen the dimeric assembly of the enzyme by improving its stability. Pharmacophore-based virtual screening allowed us to identify other possible compounds that could stabilize GALT by targeting the same pocket at the dimeric interface of the enzyme. A preliminary analysis of the effects of the compounds on the activity of the mutant form p.Gln188Arg of the GALT enzyme confirmed that, in line with computational predictions, ciclopirox and DB12882 (ombrabulin) are the compounds most capable of raising the activity of the mutant enzyme. Moreover, an in vitro cell system showed that these compounds can decrease G1P accumulation, thereby improving galactose metabolism. Nevertheless, further studies will be needed to confirm the actual stabilization of the protein structure and the role that the X site may play in raising the activity of the enzyme. However, these preliminary data underscore that the development of PCs may be an additional tool to pursue, including by drug repurposing approaches, to improve the quality of life of the patients affected by this metabolic disease.

## Figures and Tables

**Table 1:** Results of focused docking simulations in site X (the putative allosteric site) of wtGALT and the mutant (p.Gln188Arg) enzyme with each drug.

	wtGALT Xa ^1^	wtGALT Xb ^1^	p.Gln188Arg Xa ^1^	p.Gln188Arg Xb ^1^
Ambroxol				
Total number of clusters	10	8	13	8
Representative result for cluster at lowest energy (predicted binding energy of the best pose—number of poses)	−8.29 kcal/mol—17 poses	−8.30 kcal/mol—19 poses	−8.14 kcal/mol —12 poses	−8.15 kcal/mol—21 poses
Representative result for cluster with higher population (predicted binding energy of the best pose—number of poses)	−7.01 kcal/mol—33 poses	−7.26 kcal/mol—35 poses	−6.90 kcal/mol—27 poses	−7.12 kcal/mol—40 poses
Pyrimethamine				
Total number of clusters	4	2	1	3
Representative result for cluster at lowest energy (predicted binding energy of the best pose—number of poses)	−6.39 kcal/mol—11 poses	−5.88 kcal/mol—62 poses	−6.69 kcal/mol—100 poses	−5.71 kcal/mol—65 poses
Representative result for cluster with higher population (predicted binding energy of the best pose—number of poses)	−5.56 kcal/mol—55 poses	Same as above	Same as above	Same as above
Ciclopirox				
Total number of clusters	2	2	1	1
Representative result for cluster at lowest energy (predicted binding energy of the best pose—number of poses)	−7.10 kcal/mol—99 poses	−7.09 kcal/mol—99 poses	−7.12 kcal/mol—100 poses	−7.09 kcal/mol—100 poses
Representative result for cluster with higher population (predicted binding energy of the best pose—number of poses)	Same as above	Same as above	Same as above	Same as above

^1^ “Xa” means the box centered on Glu40 of chain A; “Xb” means the box centered on Glu40 of chain B.

## Data Availability

The original contributions presented in this study are included in the article/Supplementary Materials. Further inquiries can be directed to the corresponding author.

## Supplementary Materials

The supporting information can be downloaded at https://www.mdpi.com/article/10.3390/ijms26030888/s1.
