# Supplementary material for: Improvement of Mutant Galactose-1-Phosphate Uridylyltransferase (GALT) Activity by FDA-Approved Pharmacochaperones: A Preliminary Study
Source: Int J Mol Sci. 2025 Jan 21;26(3):888. doi: 10.3390/ijms26030888 (PMC11816840; doi:10.3390/ijms26030888)
Supplement: Supplementary file 1 [file ijms-26-00888-s001.zip › Supplementary_final_for-IJMS.pdf]

Improvement of mutant galactose-1 phosphate uridylyltransferase (GALT) activity by FDA-approved pharmacochaperones: a preliminary study

Bernardina Scafuri[1], Stefania Piscosquito[1], Giulia Giliberti[1], Angelo Facchiano[2], Jaden Miner[3], Bijina Balakrishnan[3], Kent Lai[3], Anna Marabotti[1]*

Supplementary Tables and Supplementary Figures

Table S1. Results of focused docking simulations between each drug and the protein known to be the target of activity as a pharmacochaperone.

	**Ambroxol vs GCase (binding site)**	**Pirimethamine vs HEX_A (binding site)**	**Ciclopirox vs UROS (allosteric site)**
Total number of clusters	7	2	5
Results for cluster at lowest energy (predicted binding energy - number of poses)	-9.07 kcal/mol - 31 poses	-9.23 kcal/mol - 90 poses	-6.17 kcal/mol - 39 poses
Results for cluster with higher population (predicted binding energy - number of poses)	Same as above	Same as above	Same as above

Table S2: Results of blind docking simulations of drugs with wtGALT

	wtGALT with both substrates	wtGALT with G1P	wtGALT with H2U	wtGALT without substrates
**Ambroxol**				
Total number of clusters	58	54	47	57
Representative result for cluster at lowest energy (predicted binding energy of the best pose - number of poses)	-8.47 kcal/mol – 6 poses	-8.88 kcal/mol – 2 poses	-8.47 kcal/mol – 5 poses	-8.54 kcal/mol – 11 poses
Representative result for cluster with higher population (predicted binding energy of the best pose - number of poses)	-7.52 kcal/mol – 7 poses	-7.93 kcal/mol – 18 poses	-7.92 kcal/mol – 18 poses	Same as above
**Pyrimethamine**				
Total number of clusters	37	37	35	33
Representative result for cluster at lowest energy (predicted binding energy of the best pose - number of poses)	-7.69 kcal/mol – 23 poses	-7.67 kcal/mol – 19 poses	-8.09 kcal/mol – 4 poses	-7.64 kcal/mol – 20 poses
Representative result for cluster with higher population (predicted binding energy of the best pose - number of poses)	Same as above	Same as above	-5.74 kcal/mol – 16 poses	Same as above
**Ciclopirox**				
Total number of clusters	37	36	35	27
Representative result for cluster at lowest energy (predicted binding energy of the best pose - number of poses)	-6.90 kcal/mol – 16 poses	-6.92 kcal/mol – 7 poses	-7.10 kcal/mol – 12 poses	-7.06 kcal/mol – 14 poses
Representative result for cluster with higher population (predicted binding energy of the best pose - number of poses)	Same as above	-6.47 kcal/mol – 17 poses	-6.88 kcal/mol – 13 poses	Same as above

Table S3: Results of blind docking simulations of each drug with mutant GALT enzyme (p.Gln188Arg).

	**p.Gln188Arg with both substrates**	**p.Gln188Arg with G1P**	**p.Gln188Arg with H2U**	**p.Gln188Arg without substrates**
**Ambroxol**				
Total number of clusters	56	58	49	66
Representative result for cluster at lowest energy (predicted binding energy of the best pose - number of poses)	-8,48 kcal/mol – 3 poses	-8.26 kcal/mol – 1 pose	-8.38 kcal/mol – 6 poses	-8.93 kcal/mol – 1 poses
Representative result for cluster with higher population (predicted binding energy of the best pose - number of poses)	-8.31 kcal/mol – 7 poses	-7.72 kcal/mol – 10 poses	-7.92 kcal/mol – 16 poses	-6.40 kcal/mol – 8 poses
**Pyrimethamine**				
Total number of clusters	36	34	35	39
Representative result for cluster at lowest energy (predicted binding energy of the best pose - number of poses)	-7.86 kcal/mol – 2 poses	-8.03 kcal/mol – 3 poses	-8.00 kcal/mol – 2 poses	-7.60 kcal/mol – 4 poses
Representative result for cluster with higher population (predicted binding energy of the best pose - number of poses)	-5.71 kcal/mol - 17 poses	-5.69 kcal/mol – 15 poses	-5.69 kcal/mol – 16 poses	-5.70 kcal/mol – 15 poses
**Ciclopirox**				
Total number of clusters	37	28	32	29
Representative result for cluster at lowest energy (predicted binding energy of the best pose - number of poses)	-6.83 kcal/mol – 10 poses	-6.90 kcal/mol – 10 poses	-7.08 kcal/mol – 13 poses	-7.08 kcal/mol – 15 poses
Representative result for cluster with higher population (predicted binding energy of the best pose - number of poses)	Same as above	-6.58 kcal/mol – 22 poses	Same as above	-6.48 kcal/mol – 22 poses

Table S4: Results of focused docking simulations in the active site A of wtGALT enzyme with each drug.

	wtGALT with G1P and H2U in site A	wtGALT with G1P in site A	wtGALT with H2U in site A	wtGALT without G1P and H2U in site A
**Ambroxol**				
Total number of clusters	18	8	12	8
Representative result for cluster at lowest energy (predicted binding energy of the best pose - number of poses)	-6.64 kcal/mol – 3 poses	-8.93 kcal/mol – 8 poses	-7.81 kcal/mol – 12 poses	-7.66 kcal/mol – 4 poses
Representative result for cluster with higher population (predicted binding energy of the best pose - number of poses)	-5.28 kcal/mol – 58 poses	-8.69 kcal/mol – 75 poses	-5.15 kcal/mol – 41 poses	-7.36 kcal/mol – 72 poses
**Pyrimethamine**				
Total number of clusters	14	2	8	5
Representative result for cluster at lowest energy (predicted binding energy of the best pose - number of poses)	-5.79 kcal/mol – 2 poses	-6.61 kcal/mol – 81 poses	-6.48 kcal/mol – 81 poses	-5.74 kcal/mol – 1 poses
Representative result for cluster with higher population (predicted binding energy of the best pose - number of poses)	-3.69 kcal/mol – 47 poses	Same as above	Same as above	-5.67 kcal/mol – 91 poses
**Ciclopirox**				
Total number of clusters	14	10	9	6
Representative result for cluster at lowest energy (predicted binding energy of the best pose - number of poses)	-7.10 kcal/mol – 22 poses	-6.86 kcal/mol – 66 poses	-7.11 kcal/mol – 7 poses	-7.15 kcal/mol – 9 poses
Representative result for cluster with higher population (predicted binding energy of the best pose - number of poses)	Same as above	Same as above	- 6.84 kcal/mol – 37 poses	-6.83 kcal/mol – 33 poses

Table S5: Results of focused docking simulations in the active site A of mutant GALT enzyme (p.Gln188Arg) with each drug.

	**p.Gln188Arg with G1P and H2U in site A**	**p.Gln188Arg with G1P in site A**	**p.Gln188Arg with H2U in site A**	**p.Gln188Arg without G1P and H2U in site A**
**Ambroxol**				
Total number of clusters	18	7	17	5
Representative result for cluster at lowest energy (predicted binding energy of the best pose - number of poses)	-6.70 kcal/mol – 4 poses	-8.56 kcal/mol – 83 pose	-7.07 kcal/mol – 5 poses	-7.24 kcal/mol – 77 poses
Representative result for cluster with higher population (predicted binding energy of the best pose - number of poses)	-5.24 kcal/mol – 56 poses	Same as above	-5.09 kcal/mol - 44 poses	Same as above
**Pyrimethamine**				
Total number of clusters	13	2	10	1
Representative result for cluster at lowest energy (predicted binding energy of the best pose - number of poses)	-5.72 kcal/mol – 3 poses	-6.48 kcal/mol – 78 poses	-6.11 kcal/mol – 61 poses	-5.64 kcal/mol – 100 poses
Representative result for cluster with higher population (predicted binding energy of the best pose - number of poses)	-3.61 kcal/mol – 53 poses	Same as above	Same as above	Same as above
**Ciclopirox**				
Total number of clusters	16	8	10	4
Representative result for cluster at lowest energy (predicted binding energy of the best pose - number of poses)	-7.12 kcal/mol – 26 poses	-6.86 kcal/mol – 68 poses	-7.01 kcal/mol – 10 poses	-7.18 kcal/mol – 9 poses
Representative result for cluster with higher population (predicted binding energy of the best pose - number of poses)	Same as above	Same as above	-6.82 kcal/mol – 36 poses	-6.88 kcal/mol – 84 poses

Table S6: Results of focused docking simulations in the central cavity of wtGALT and mutant (p.Gln188Arg) enzyme with each drug.

	**wtGALT with substrates**	**wtGALT without substrates**	**p.Gln188Arg with substrates**	**p.Gln188Arg without substrates**
**Ambroxol**				
Total number of clusters	27	29	23	27
Representative result for cluster at lowest energy (predicted binding energy of the best pose - number of poses)	-7.45 kcal/mol – 14 poses	-7.64 kcal/mol – 9 pose	-7.69 kcal/mol – 1 poses	-7.61 kcal/mol – 5 poses
Representative result for cluster with higher population (predicted binding energy of the best pose - number of poses)	Same as above	-7.39 kcal/mol – 11 poses	-6.96 kcal/mol – 14 poses	-7.28 kcal/mol – 10 poses
**Pyrimethamine**				
Total number of clusters	2	2	2	2
Representative result for cluster at lowest energy (predicted binding energy of the best pose - number of poses)	-6.88 kcal/mol – 76 poses	-6.85 kcal/mol – 70 poses	-6.70 kcal/mol – 73 poses	-6.85 kcal/mol – 78 poses
Representative result for cluster with higher population (predicted binding energy of the best pose - number of poses)	Same as above	Same as above	Same as above	Same as above
**Ciclopirox**				
Total number of clusters	8	10	5	8
Representative result for cluster at lowest energy (predicted binding energy of the best pose - number of poses)	-7.13 kcal/mol – 75 poses	-7.12 kcal/mol – 74 poses	-7.13 kcal/mol – 88 poses	-7.12 kcal/mol – 77 poses
Representative result for cluster with higher population (predicted binding energy of the best pose - number of poses)	Same as above	Same as above	Same as above	Same as above

Legends to Supplementary Figures

Figure S1: interactions between wtGALT (left panels) with selected drugs, or between p.Gln188Arg enzyme (right panels) with selected drugs, identified by blind docking. Panels (a) and (b) show the interactions of ambroxol; panels (c) and (d) the interactions of pyrimethamine; panels (e) and (f) the interactions of ciclopirox. The drugs are represented in CPK modes and colored according to the different simulations: cyan represents simulation with both ligands in the active sites; magenta represents simulations with only G1P in the active sites; orange represents simulations with only H2U in the active sites; green represents simulations with no substrates in the active sites. The darker shades indicate those poses with better interaction energy; the lighter shades indicate those poses representing highly populated clusters. The substrates are represented in stick mode and colored black, the Zn ion as a sphere and colored grey.

Figure S2: interactions between wtGALT and selected drugs, identified by docking focused on active site A. Panel (a): docking made in the presence of both substrates in the active site A; Panel (b): docking made in the presence of G1P in the active site A; Panel (c): docking made in the presence of H2U in the active site A; Panel (d): docking made in the absence of both substrates in the active site A. The three drugs are represented in CPK mode and colored cyan (ambroxol), yellow (pyrimethamine) and magenta (ciclopirox); the darker shades indicate those poses with better interaction energy; the lighter shades indicate those poses representing highly populated clusters. The substrates are represented in stick mode and colored black, the Zn ion as a sphere and colored grey.

Figure S3: interactions between p.Gln188Arg and selected drugs, identified by docking focused on active site A. Panel (a): docking made in the presence of both substrates in the active site A; Panel (b): docking made in the presence of G1P in the

active site A; Panel (c): docking made in the presence of H2U in the active site A; Panel (d): docking made in the absence of both substrates in the active site A. The three drugs are represented in CPK mode and colored cyan (ambroxol), yellow (pyrimethamine) and magenta (ciclopirox); the darker shades indicate those poses with better interaction energy; the lighter shades indicate those poses representing highly populated clusters. The substrates are represented in stick mode and colored black, the Zn ion as a sphere

Figure S4: interactions between wtGALT (left panels) with selected drugs, or between p.Gln188Arg enzyme (right panels) with selected drugs, identified by docking focused on the central cavity of the enzyme. Panels (a) and (b) show the docking simulations made in the presence of both substrates in the active sites; panels (c) and (d) show the docking simulations made in the absence of substrates in the active site. The three drugs are represented in CPK mode and colored cyan (ambroxol), yellow (pyrimethamine) and magenta (ciclopirox); the darker shades indicate those poses with better interaction energy; the lighter shades indicate those poses representing highly populated clusters. The substrates are represented in stick mode and colored black, the Zn ion as a sphere and colored grey.

Figure S5: **Detailed interactions between ambroxol and wtGALT or p.Gln188Arg enzyme identified by docking focused on the putative allosteric site (site "X")**. Panel (a): result of docking on site Xa on wtGALT, pose with best energy. Panel (b): result of docking on site Xa on wtGALT, pose representing the most populated cluster. Panel (c): result of docking on site Xa on p.Gln188Arg, pose with best energy. Panel (d): result of docking on site Xa on p.Gln188Arg, pose representing the most populated cluster. Panel (e): result of docking on site Xb on wtGALT, pose with best energy. Panel (f): result of docking on site Xb on wtGALT, pose representing the most populated cluster. Panel (g):

result of docking on site Xb on p.Gln188Arg, pose with best energy. Panel (h): result of docking on site Xa on p.Gln188Arg, pose representing the most populated cluster.

Figure S6: Detailed interactions between pyrimethamine and wtGALT or p.Gln188Arg enzyme identified by docking focused on the putative allosteric site (site "X"). Panel (a): result of docking on site Xa on wtGALT, pose with best energy. Panel (b): result of docking on site Xa on wtGALT, pose representing the most populated cluster. Panel (c): result of docking on site Xa on p.Gln188Arg (the pose with best energy is also representative of the most populated cluster). Panel (d): result of docking on site Xb on wtGALT (the pose with best energy is also representative of the most populated cluster). Panel (e): result of docking on site Xb on p.Gln188Arg (the pose with best energy is also representative of the most populated cluster).

Figure S7: Detailed interactions between DB06306 and wtGALT or p.Gln188Arg enzyme identified by docking focused on the putative allosteric site (site "X"). Panel (a): result of docking on site Xa on wtGALT (the pose with best energy is also representative of the most populated cluster). Panel (b): result of docking on site Xb on wtGALT, pose with best energy. Panel (c): result of docking on site Xb on wtGALT, pose representing the most populated cluster. Panel (d): result of docking on site Xa on p.Gln188Arg (the pose with best energy is also representative of the most populated cluster). Panel (e): result of docking on site Xb on p.Gln188Arg, pose with best energy. Panel (f): result of docking on site Xb on wtGALT, pose representing the most populated cluster.

Figure S8: Detailed interactions between DB08875 and wtGALT or p.Gln188Arg enzyme identified by docking focused on the putative allosteric site (site "X"). Panel (a): result of docking on site Xa on wtGALT. Panel (b): result of docking on site Xb on

wtGALT. Panel (c): result of docking on site Xa on p.Gln188Arg; Panel (d): result of docking on site Xb on p.Gln188Arg.

Figure S9: Detailed interactions between DB13403 and wtGALT or p.Gln188Arg enzyme identified by docking focused on the putative allosteric site (site "X"). Panel (a): result of docking on site Xa on wtGALT. Panel (b): result of docking on site Xb on wtGALT. Panel (c): result of docking on site Xa on p.Gln188Arg; Panel (d): result of docking on site Xb on p.Gln188Arg.

Figure S10: Detailed interactions between DB14002 and wtGALT or p.Gln188Arg enzyme identified by docking focused on the putative allosteric site (site "X"). Panel (a): result of docking on site Xa on wtGALT, pose with best energy. Panel (b): result of docking on site Xa on wtGALT, pose representative of the most populated cluster. Panel (c): result of docking on site Xb on wtGALT (the pose with best energy is also representative of the most populated cluster). Panel (d): result of docking on site Xa on p.Gln188Arg, pose with best energy. Panel (e): result of docking on site Xa on p.Gln188Arg, pose representative of the most populated cluster. Panel (f): result of docking on site Xb on p.Gln188Arg (the pose with best energy is also representative of the most populated cluster).

Figure S1

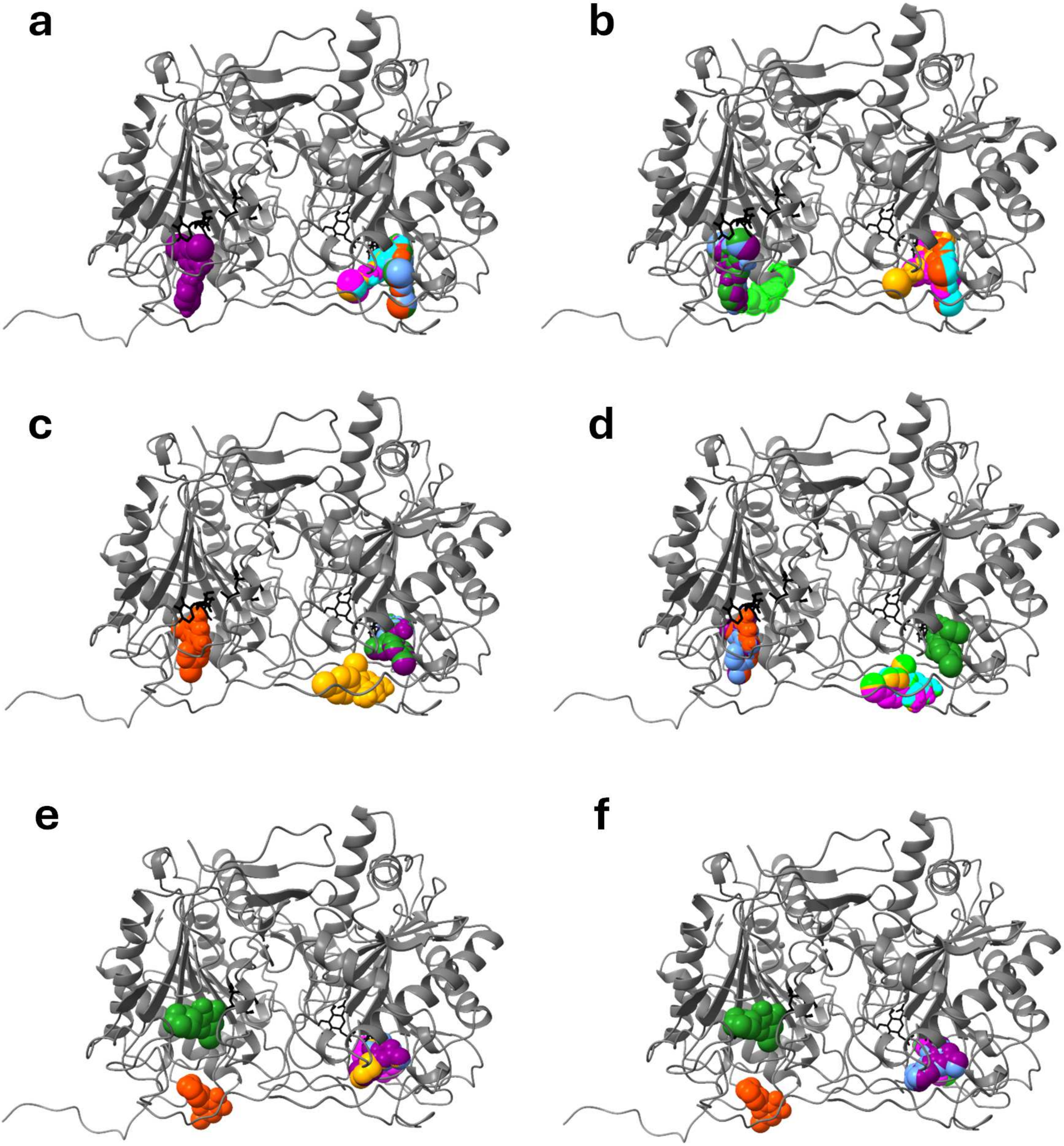

Figure S2

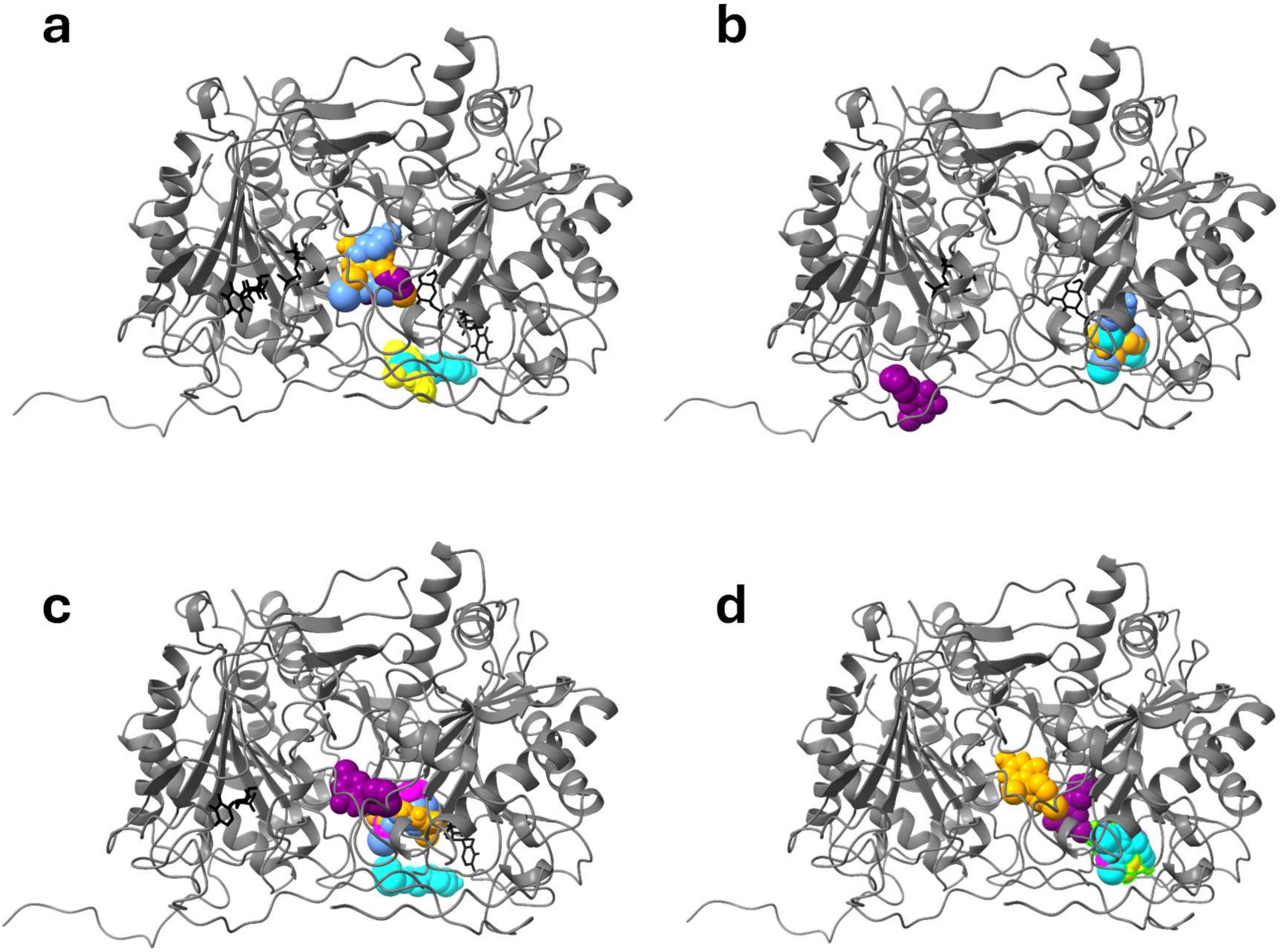

Figure S3

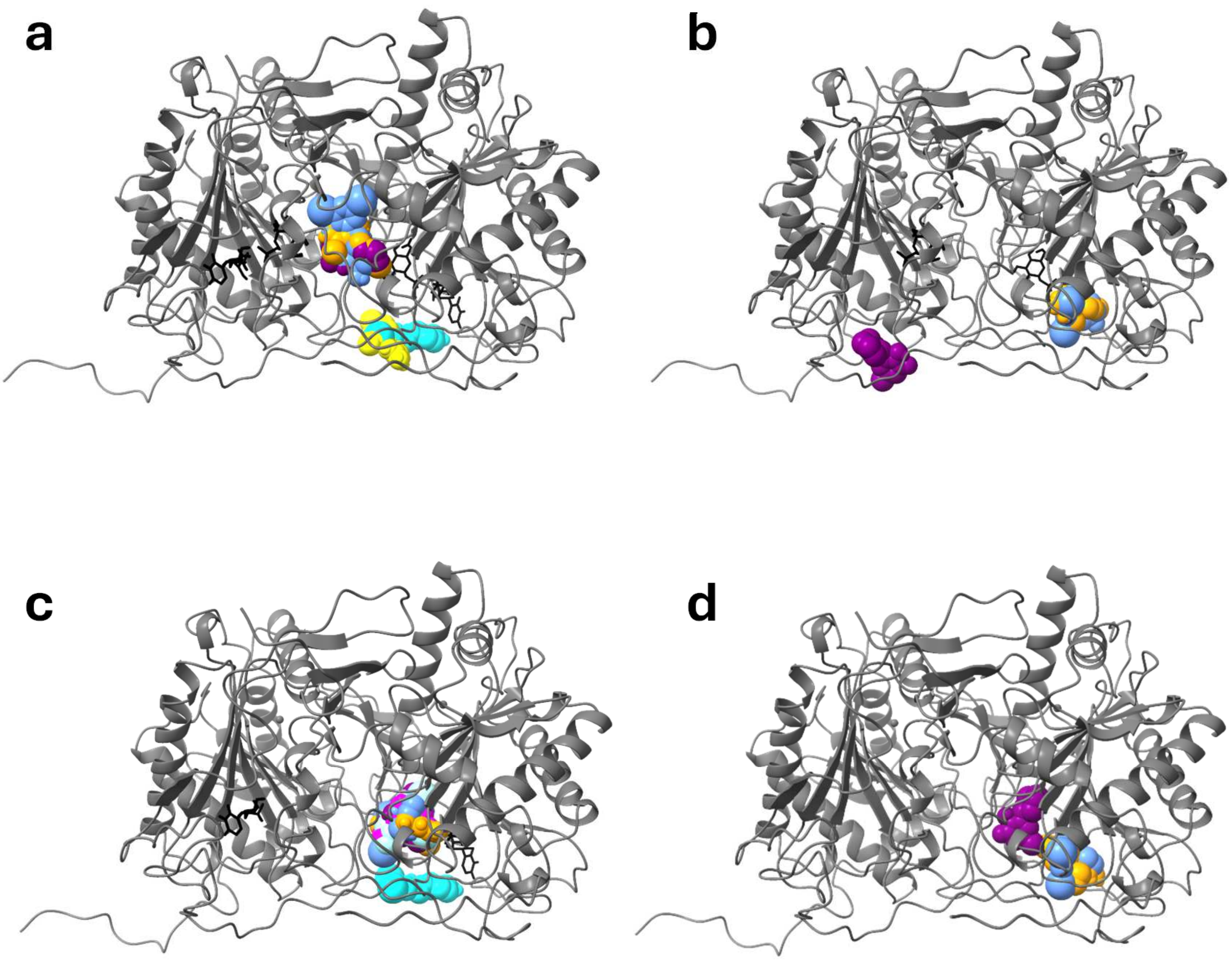

Figure S4

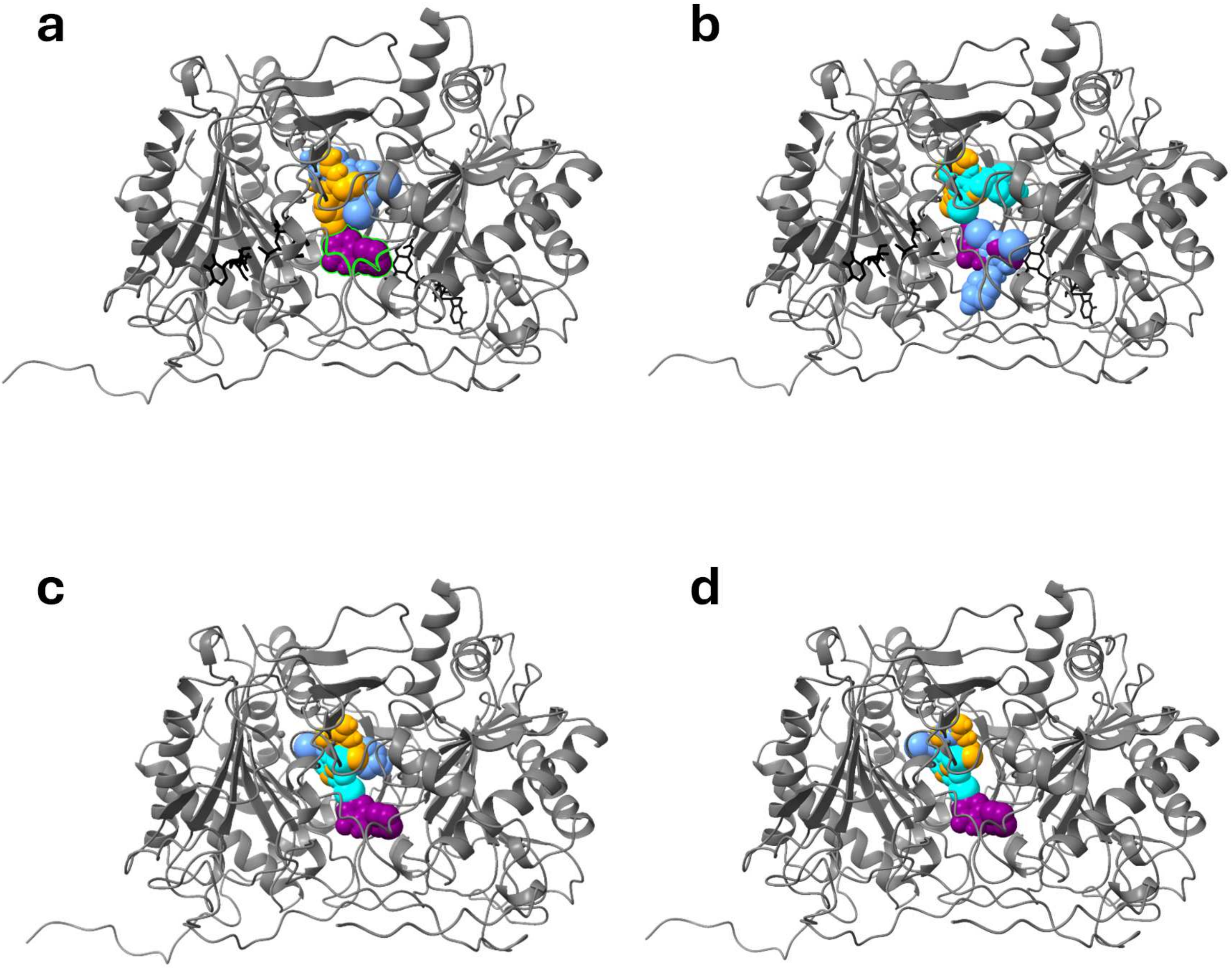

Figure S5

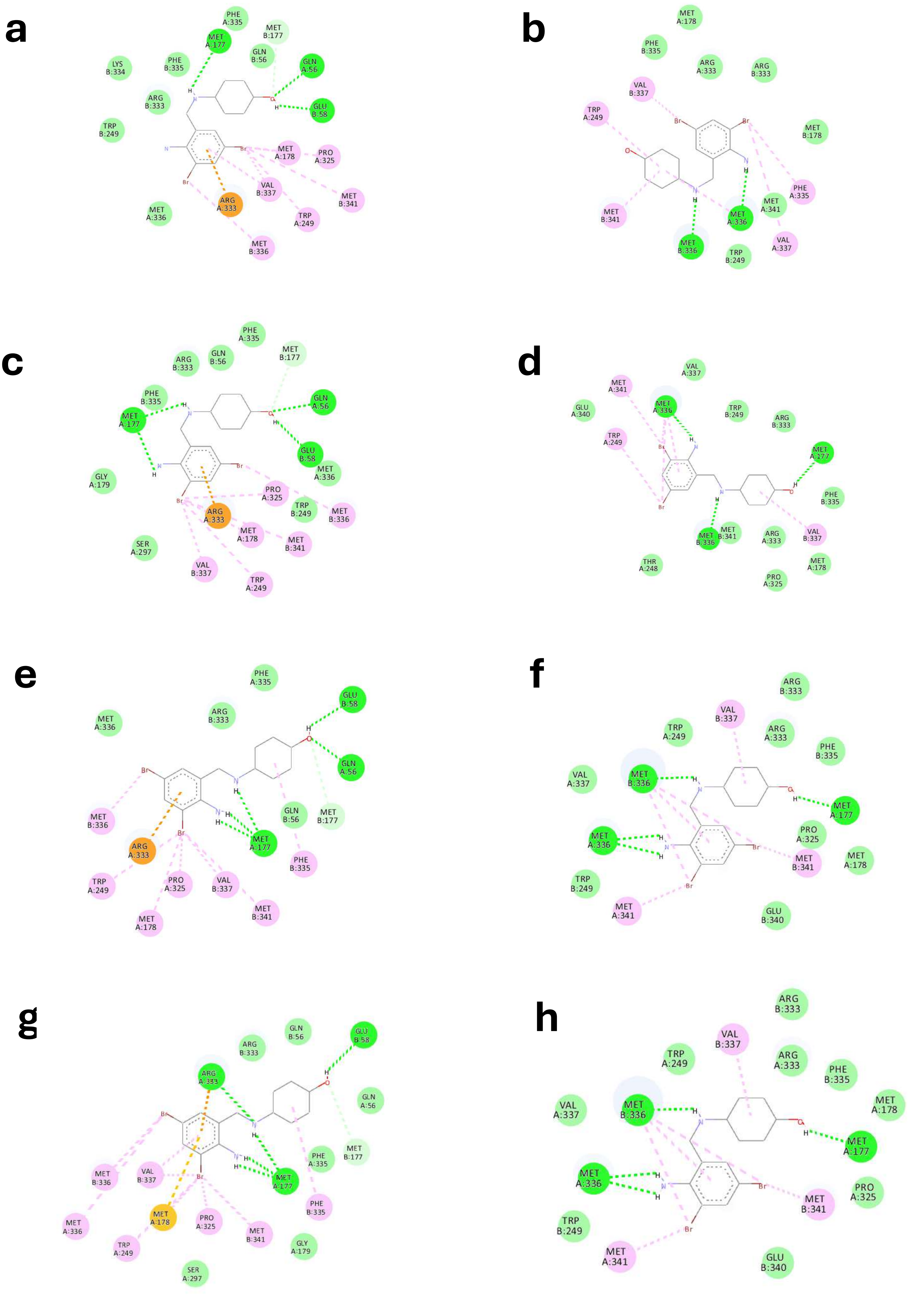

a
PHE A:335
MET A:177
MET B:177
GLN B:56
GLN A:56
LYS B:334
PHE B:335
ARG B:333
GLU B:58
TRP B:249
MET A:178
PRO A:325
VAL B:337
MET B:341
MET A:336
ARG A:333
TRP A:249
MET B:336
b
MET A:178
PHE B:335
ARG A:333
ARG B:333
VAL B:337
TRP A:249
MET B:178
PHE A:335
MET A:341
MET B:341
MET A:336
VAL A:337
MET B:336
TRP B:249
c
PHE A:335
ARG B:333
GLN B:56
MET B:177
PHE B:335
GLN A:56
MET A:177
GLU B:58
MET A:336
GLY A:179
PRO A:325
TRP B:249
MET B:336
ARG A:333
MET A:178
MET B:341
SER A:297
VAL B:337
TRP A:249
d
VAL A:337
MET A:341
MET A:336
GLU A:340
TRP B:249
ARG B:333
TRP A:249
MET A:177
PHE B:335
MET B:336
MET B:341
ARG A:333
VAL B:337
THR A:248
MET A:178
PRO A:325
e
PHE A:335
GLU B:58
MET A:336
ARG B:333
GLN A:56
MET B:336
GLN B:56
MET B:177
MET A:177
ARG A:333
PHE B:335
TRP A:249
PRO A:325
VAL B:337
MET A:178
MET B:341
f
ARG B:333
VAL B:337
TRP A:249
ARG A:333
PHE B:335
VAL A:337
MET B:336
MET A:177
MET A:336
PRO A:325
MET B:341
MET A:178
TRP B:249
MET A:341
GLU B:340
g
GLN B:56
GLU B:58
ARG B:333
ARG A:333
GLN A:56
PHE A:335
MET B:177
MET B:336
VAL B:337
MET A:177
PHE B:335
MET A:336
MET A:178
PRO A:325
MET B:341
GLY A:179
TRP A:249
SER A:297
h
ARG B:333
VAL B:337
TRP A:249
ARG A:333
PHE B:335
MET A:178
VAL A:337
MET B:336
MET A:177
MET A:336
PRO A:325
MET B:341
TRP B:249
MET A:341
GLU B:340

Figure S6

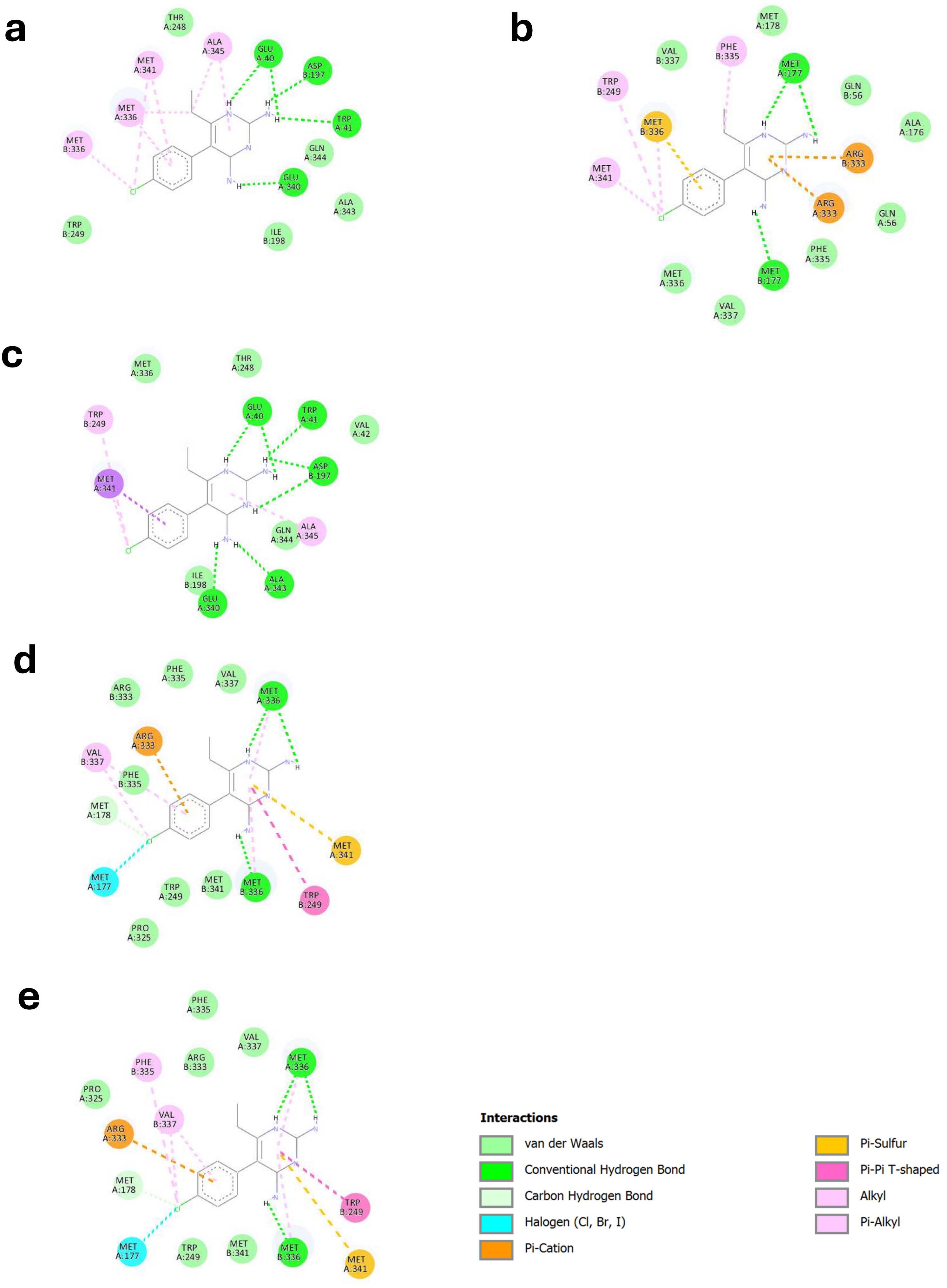
a
THR A:248
ALA A:345
GLU A:40
ASP B:197
MET A:341
MET A:336
TRP A:41
MET B:336
GLN A:344
GLU A:340
ALA A:343
TRP B:249
ILE B:198
b
MET A:178
VAL B:337
PHE B:335
MET A:177
GLN B:56
TRP B:249
MET B:336
ALA A:176
ARG B:333
MET A:341
ARG A:333
GLN A:56
PHE A:335
MET A:336
MET B:177
VAL A:337
c
MET A:336
THR A:248
GLU A:40
TRP A:41
VAL A:42
TRP B:249
ASP B:197
MET A:341
GLN A:344
ALA A:345
ILE B:198
ALA A:343
GLU A:340
d
PHE A:335
VAL A:337
ARG B:333
MET A:336
ARG A:333
VAL B:337
PHE B:335
MET A:178
MET A:341
MET A:177
TRP A:249
MET B:341
MET B:336
TRP B:249
PRO A:325
e
PHE A:335
VAL A:337
PHE B:335
ARG B:333
MET A:336
PRO A:325
VAL B:337
ARG A:333
MET A:178
TRP B:249
MET A:177
TRP A:249
MET B:341
MET B:336
MET A:341
Interactions
van der Waals
Conventional Hydrogen Bond
Carbon Hydrogen Bond
Halogen (Cl, Br, I)
Pi-Cation
Pi-Sulfur
Pi-Pi T-shaped
Alkyl
Pi-Alkyl

Figure S7

a

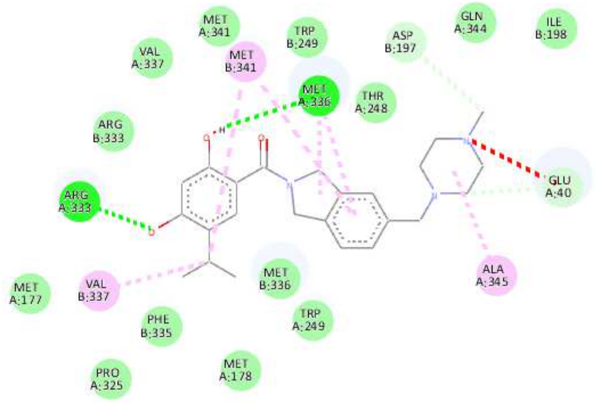

b

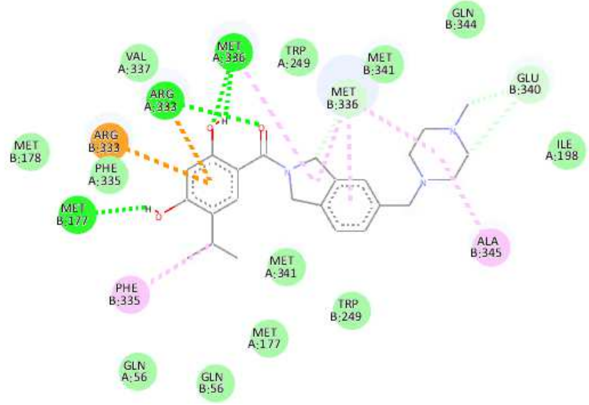

c

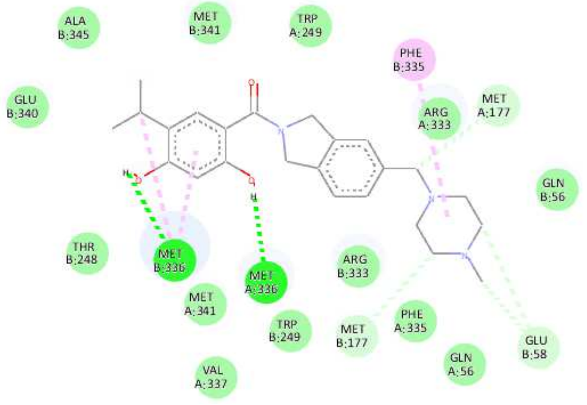

d

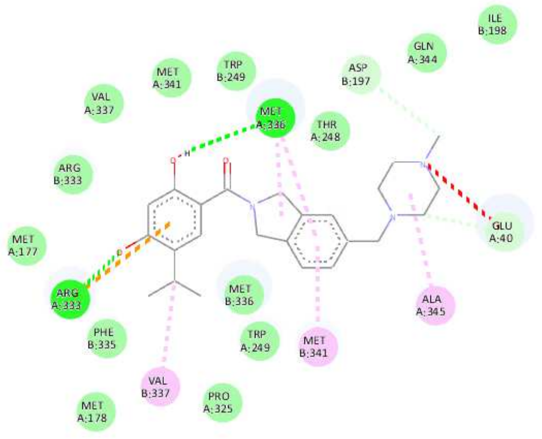

e

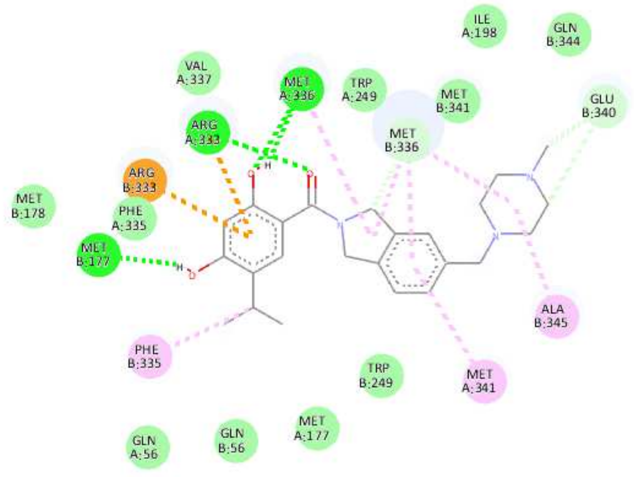

f

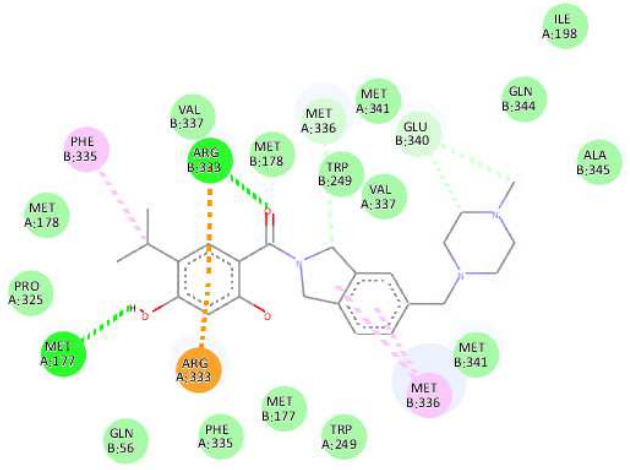

Figure S8

a

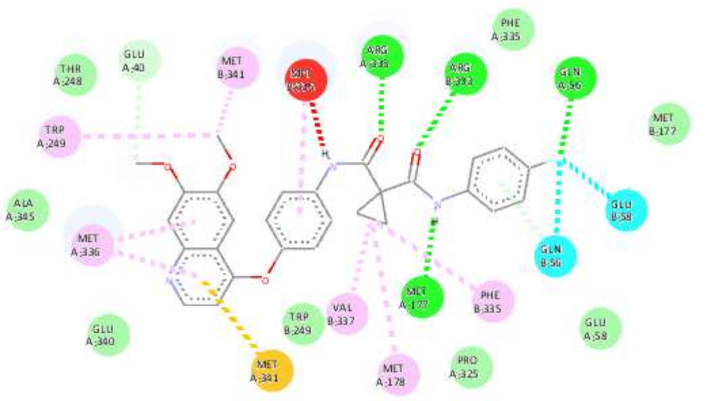

b

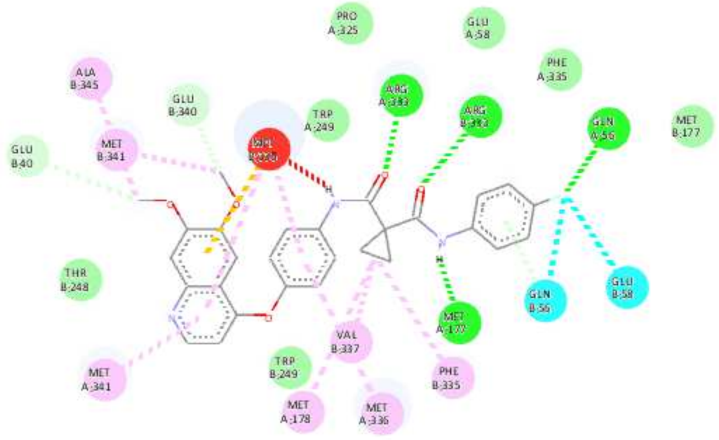

c

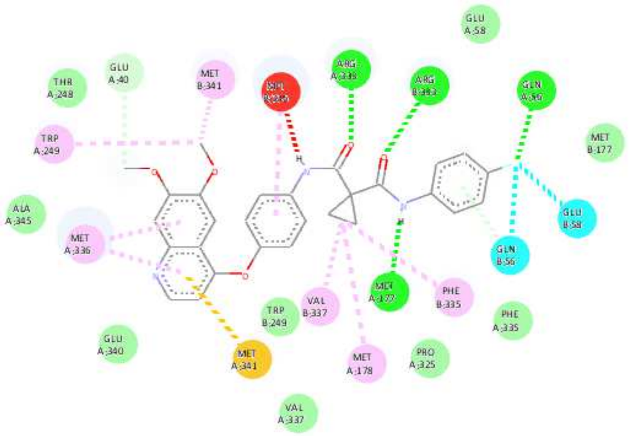

d

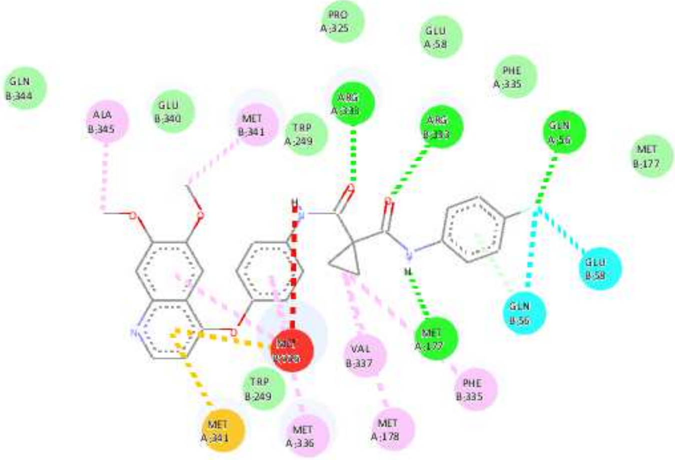

Figure S9

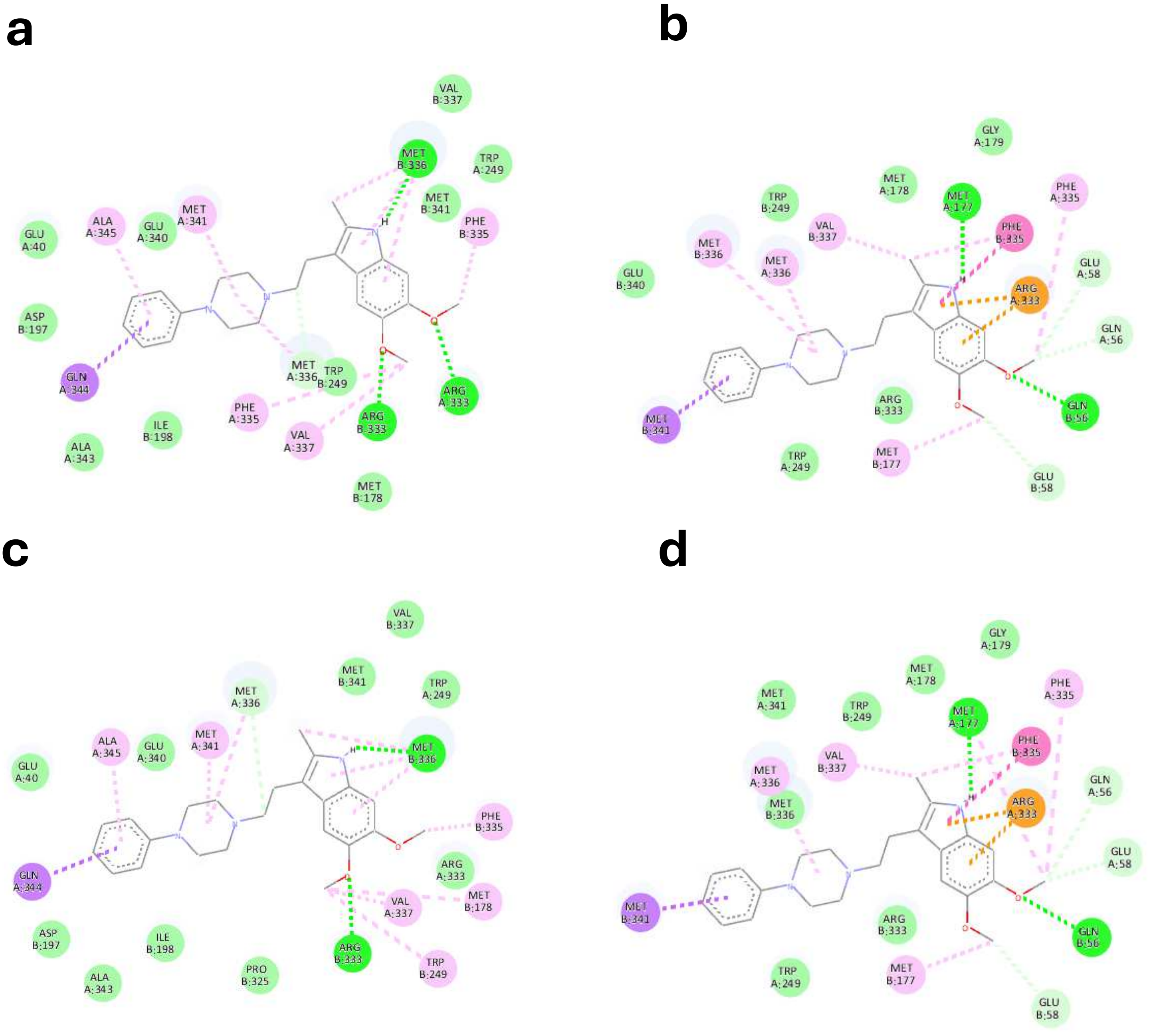
a
VAL B:337
MET B:336
TRP A:249
MET B:341
PHE B:335
GLU A:40
ALA A:345
GLU A:340
MET A:341
ASP B:197
GLN A:344
MET A:336
TRP B:249
PHE A:335
ILE B:198
ALA A:343
VAL A:337
ARG B:333
ARG A:333
MET B:178
b
GLY A:179
MET A:178
TRP B:249
MET A:177
PHE A:335
VAL B:337
PHE B:335
MET B:336
MET A:336
GLU B:340
GLU A:58
ARG A:333
GLN A:56
MET B:341
ARG B:333
GLN B:56
TRP A:249
MET B:177
GLU B:58
c
VAL B:337
MET B:341
TRP A:249
MET A:336
ALA A:345
GLU A:340
MET A:341
GLU A:40
MET B:336
PHE B:335
GLN A:344
ARG A:333
MET B:178
VAL A:337
ASP B:197
ILE B:198
ARG B:333
TRP B:249
ALA A:343
PRO B:325
d
GLY A:179
MET A:178
MET A:341
TRP B:249
MET A:177
PHE A:335
PHE B:335
VAL B:337
MET A:336
MET B:336
GLN A:56
ARG A:333
GLU A:58
MET B:341
ARG B:333
GLN B:56
TRP A:249
MET B:177
GLU B:58

Figure S10

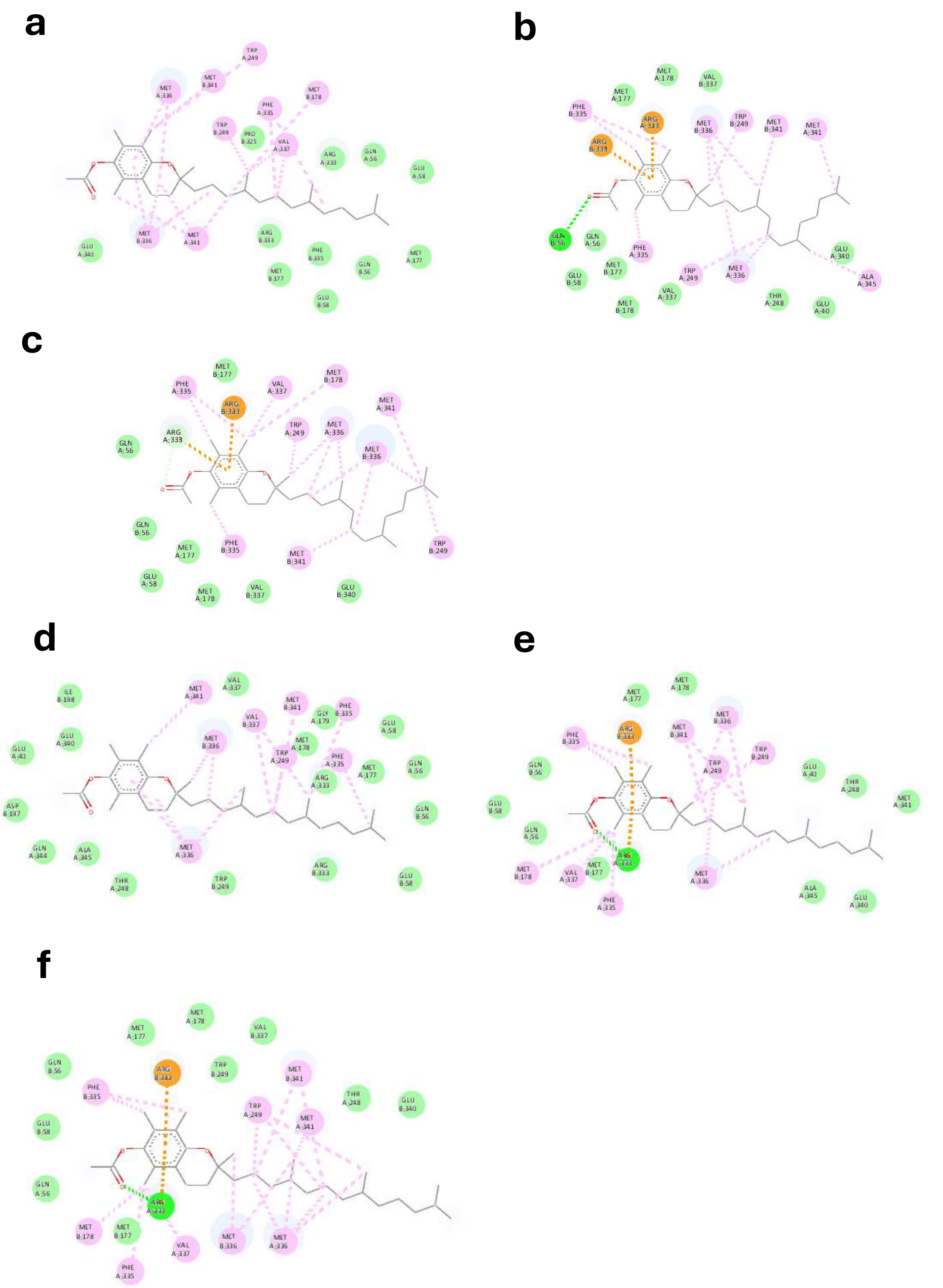
a
TRP A:249
MET A:336
MET B:341
MET B:178
PHE A:335
TRP B:249
PRO B:325
VAL A:337
ARG A:333
GLN A:56
GLU A:58
MET B:336
MET A:341
ARG B:333
GLU A:340
PHE B:335
GLN B:56
MET A:177
MET B:177
GLU B:58
b
MET A:178
VAL B:337
MET A:177
PHE B:335
ARG A:333
ARG B:333
MET B:336
TRP B:249
MET B:341
MET A:341
GLN B:56
GLN A:56
PHE A:335
MET B:177
GLU B:58
TRP A:249
MET A:336
GLU A:340
ALA A:345
VAL A:337
THR A:248
GLU A:40
MET B:178
c
MET B:177
PHE A:335
VAL A:337
MET B:178
ARG B:333
MET A:341
ARG A:333
TRP A:249
MET A:336
GLN A:56
MET B:336
GLN B:56
PHE B:335
MET A:177
MET B:341
TRP B:249
GLU A:58
MET A:178
VAL B:337
GLU B:340
d
VAL A:337
ILE B:198
MET A:341
MET B:341
GLY A:179
PHE B:335
GLU A:340
VAL B:337
MET B:336
GLU A:58
GLU A:40
MET A:178
TRP A:249
PHE A:335
MET A:177
GLN A:56
ARG A:333
ASP B:197
GLN B:56
GLN A:344
ALA A:345
MET A:336
THR A:248
TRP B:249
ARG B:333
GLU B:58
e
MET A:178
MET A:177
MET B:336
ARG B:333
MET B:341
PHE B:335
TRP B:249
GLN B:56
TRP A:249
GLU A:40
THR A:248
GLU B:58
MET A:341
GLN A:56
ARG A:333
MET B:178
VAL A:337
MET B:177
MET A:336
ALA A:345
GLU A:340
PHE A:335
f
MET A:178
MET A:177
VAL B:337
GLN B:56
ARG B:333
TRP B:249
MET B:341
PHE B:335
THR A:248
GLU B:340
TRP A:249
MET A:341
GLU B:58
GLN A:56
ARG A:333
MET B:178
MET B:177
VAL A:337
MET B:336
MET A:336
PHE A:335
